# Temporal transcriptomic profiling uncovers multilevel suppression of RANKL-driven osteoclast differentiation by 14-3-3ζ

**DOI:** 10.1016/j.jbc.2026.113473

**Published:** 2026-08-24

**Authors:** Rathinam Ayyasamy, Jaya Bhandari, Kirti Parwani, Hope LeeAnn York, Jenna McGowan, Saurabh Chattopadhyay, Ritu Chakravarti

**Affiliations:** 1 Department of Internal Medicine, University of Kentucky, Lexington, Kentucky, USA; 2 Department of Physiology & Pharmacology, University of Toledo, Toledo, Ohio, USA; 3 Department of Microbiology, Immunology, & Molecular Genetics, University of Kentucky, Lexington, Kentucky, USA

**Keywords:** 14-3-3ζ, Lgr4, osteoclast, Ptpn11, RANKL, RNA-seq, Ywhaz

## Abstract

Receptor activator of nuclear factor κB ligand (RANKL) is the central driver of osteoclast differentiation and function, yet the regulatory checkpoints that restrain RANKL signaling remain incompletely defined. We previously demonstrated that 14-3-3ζ (*Ywhaz*) suppresses RANKL signaling by targeting TRAF6 at the post-translational level. Here, we identify a previously unrecognized transcriptional mechanism by which 14-3-3ζ constrains RANKL-induced osteoclastogenesis. Using an unbiased, time-resolved transcriptomic analysis (0–40 h) in primary rat bone marrow-derived preosteoclasts, we show that loss of 14-3-3ζ augments RANKL-induced expression of canonical osteoclast genes (*Acp5, Ctsk*, *Car2*), confirming its suppressive role in osteoclast differentiation. Beyond core osteoclast markers, 14-3-3ζ deficiency enhanced the induction of chemokines (*Ccl7*), focal adhesion (*Rac2*), and lysosomal markers (*Lamp1, CTSZ*), identifying its role in regulating the amplification of RANKL signaling. Mechanistically, we observed that 14-3-3ζ targets both positive and negative regulators of RANKL. While 14-3-3ζ promoted *Lgr4*, an inhibitor of RANKL signaling and NFATc1 expression, it concurrently suppressed SHP-2 (*Ptpn11*) expression, a phosphatase that promotes cell-cell fusion, a critical late step in osteoclast formation. Inhibition of SHP-2 activity with a specific pharmacological inhibitor abolished osteoclast differentiation in 14-3-3ζ-deficient cells, establishing SHP-2 as a functional effector downstream of 14-3-3ζ. Collectively, these findings reveal that 14-3-3ζ exerts dual transcriptional control over RANKL signaling, limiting both early osteoclastogenic gene induction and late-stage cell fusion. Loss of this regulatory brake increases susceptibility to RANKL-induced osteoclastogenesis, providing a mechanistic basis for enhanced bone loss, and highlighting 14-3-3ζ as a potential therapeutic target in inflammatory bone disorders.

14-3-3ζ is a member of 14-3-3 family of proteins (1). Like other isoforms, 14-3-3ζ is a critical adaptor protein involved in diverse cellular signaling pathways. It interacts with a broad range of signaling intermediates, including kinases, phosphatases, and both phosphorylated and non-phosphorylated proteins (2, 3). It also participates in sub-cellular trafficking of signaling intermediates and transcription factors (4). Altered expression of 14-3-3ζ has been implicated in multiple human autoimmune diseases, including large vessel vasculitis, type 1 diabetes, and rheumatoid arthritis (5, 6, 7, 8). Genetic variants in 14-3-3ζ are associated with Cardiofaciocutaneous syndrome, suggesting a vital role for 14-3-3ζ in musculoskeletal and neuronal development (9).

RANKL (Receptor Activator of Nuclear Factor-κB Ligand, gene: *TNFSF11*) is the principal cytokine driving osteoclast differentiation and activation (10). It is secreted by osteocytes, osteoblasts, and T cells during bone remodeling and inflammation. RANKL signals through its receptor RANK, expressed on pre-osteoclasts-macrophages primed for osteoclast lineage commitment in the presence of macrophage colony-stimulating factor (M-CSF) (11). Upon RANKL binding, RANK recruits TRAF6 and various kinases, initiating signaling cascades that activate transcription factors such as NFATc1, AP-1, NF-κB, and MITF. The nuclear translocation of these factors promotes the expression of genes essential for osteoclast differentiation and function (12).

Osteoclast differentiation is a multi-step process that typically spans 3 to 11 days and involves temporally regulated gene expression. Time-course analyses have shown that M-CSF and RANKL exert synergistic effects on osteoclastogenesis, with the most pivotal transcriptional events occurring during the early phase of differentiation (13). RANKL-induced transcriptional changes are transient and time-dependent, involving a range of genes related to cytokines, growth factors, intracellular trafficking, cell adhesion/motility, and transcriptional regulation (14, 15). Due to its significant effects on bone health, RANKL signaling is tightly regulated by both positive and negative regulators (16). LGR4 (*LGR4*) is one such negative regulator, whereas SHP-2 (*PTPN11*) is positive regulator of RANKL signaling (16, 17). RANKL stimulation induces 14-3-3ζ expression 24 h after treatment, followed by a decline, suggesting a temporal regulatory role (18).

We previously demonstrated that 14-3-3ζ suppresses inflammatory arthritis in rodent models and significantly affects bone quality. Lewis rats deficient in 14-3-3ζ (*Ywhaz*^KO^) exhibited a marked reduction in both bone mass and bone mineral density under inflammatory arthritis models (7). The mechanistic studies demonstrated that 14-3-3ζ negatively regulates M-CSF/RANKL-induced osteoclastogenesis *ex vivo*. Specifically, macrophages deficient in 14-3-3ζ differentiated into larger and more active osteoclasts, compared to WT controls, underscoring the inhibitory role of 14-3-3ζ in osteoclast formation and bone loss (7, 19). Anti-inflammatory therapies targeting inflammatory arthritis in animals have indicated a significant therapeutic effect of 14-3-3ζ on bone quality (7, 20). In the current study, we investigated the temporal regulation of RANKL-induced transcriptional responses by 14-3-3ζ through a time-course analysis during osteoclast differentiation. Our results using bulk RNA-Seq, as described below, provide insights into transcriptional regulation by which 14-3-3ζ dampens RANKL-induced osteoclastic events.

## Results

We performed bulk RNA sequencing of bone marrow-derived macrophages (BMDM) from WT and *Ywhaz*^KO^ male Lewis rats to identify the molecular mechanisms underlying the role of 14-3-3ζ in regulating inflammation-induced bone loss. Since RANKL-induced osteoclastogenesis occurs in a temporally regulated manner (11), a time-course experiment allows us to understand the stage-specific effects of 14-3-3ζ on gene transcription. The *Ywhaz*^KO^ showed a comparable SIRPA^+^ macrophage population in isolated BMDM, and the numbers of cells pre- and post-RANKL stimulation were similar to those in WT (Fig. S1, *B* and *C*).

To evaluate sample variability and ensure consistent results across replicates, we first analyzed pairwise sample correlations and performed principal component analysis, hierarchical clustering heatmaps of differentially expressed genes (DEGs), and Venn diagram comparisons of DEGs between WT and *Ywhaz*^KO^ BMDM treated with or without M-CSF/RANKL (Fig. S1, *D*–*G*). Based on the Principal component analysis, in contrast to other biological replicates, which formed tight and well-separated clusters, two samples (KO_A40 and WT_C40) segregated distinctly from their respective groups and fell outside the variance defined by replicate distributions in principal component space. However, the results below include all samples; no exclusion was performed.

### Loss of 14-3-3ζ impacts inflammation and cellular adhesion in the absence of any stimulation

Since *Ywhaz*^KO^ animals exhibit increased susceptibility to inflammation, the transcriptomes of unstimulated WT and *Ywhaz*^KO^ BMDM were compared to identify basal-level differences. The Venn diagram showed 196 uniquely expressed genes in *Ywhaz*^KO^ BMDM (Fig. 1*A*). A volcano plot showed 1310 genes were significantly upregulated in the *Ywhaz*^KO^ group, while fewer genes were significantly downregulated (Fig. 1*B*). As expected, *Ywhaz* was one of the highly ranked downregulated genes in the *Ywhaz*^KO^. The heatmap of top 50 DEGs included several inflammatory molecules (*S100a6*), integrins (*Itga6*) and phosphatases (*Ptpn11*) (Fig. 1*C*). Enrichment analysis of DEGs revealed altered gene networks related to endocytosis, MAPK signaling, Rap1 signaling, focal adhesion, and chemokine signaling (Fig. 1*D*). It is relevant as 14-3-3ζ is a known adaptor of MAP kinases and participates in several signal transductions involving Ras-MAPK pathways. Reactome pathway analysis revealed roles in platelet activation, signaling, and degranulation, consistent with previous findings that 14-3-3ζ binds and regulates Ca2^+^-activated cytoskeletal factors (Figs. 1*E* and S2, *A*–*C*) (21). The affected pathways are associated with clinical disorders such as Noonan syndrome, aneurysms, and rasopathies (Fig. 1*F*). These changes provide insight into how 14-3-3ζ impacts the overall inflammatory status in macrophages.

**Figure 1:**
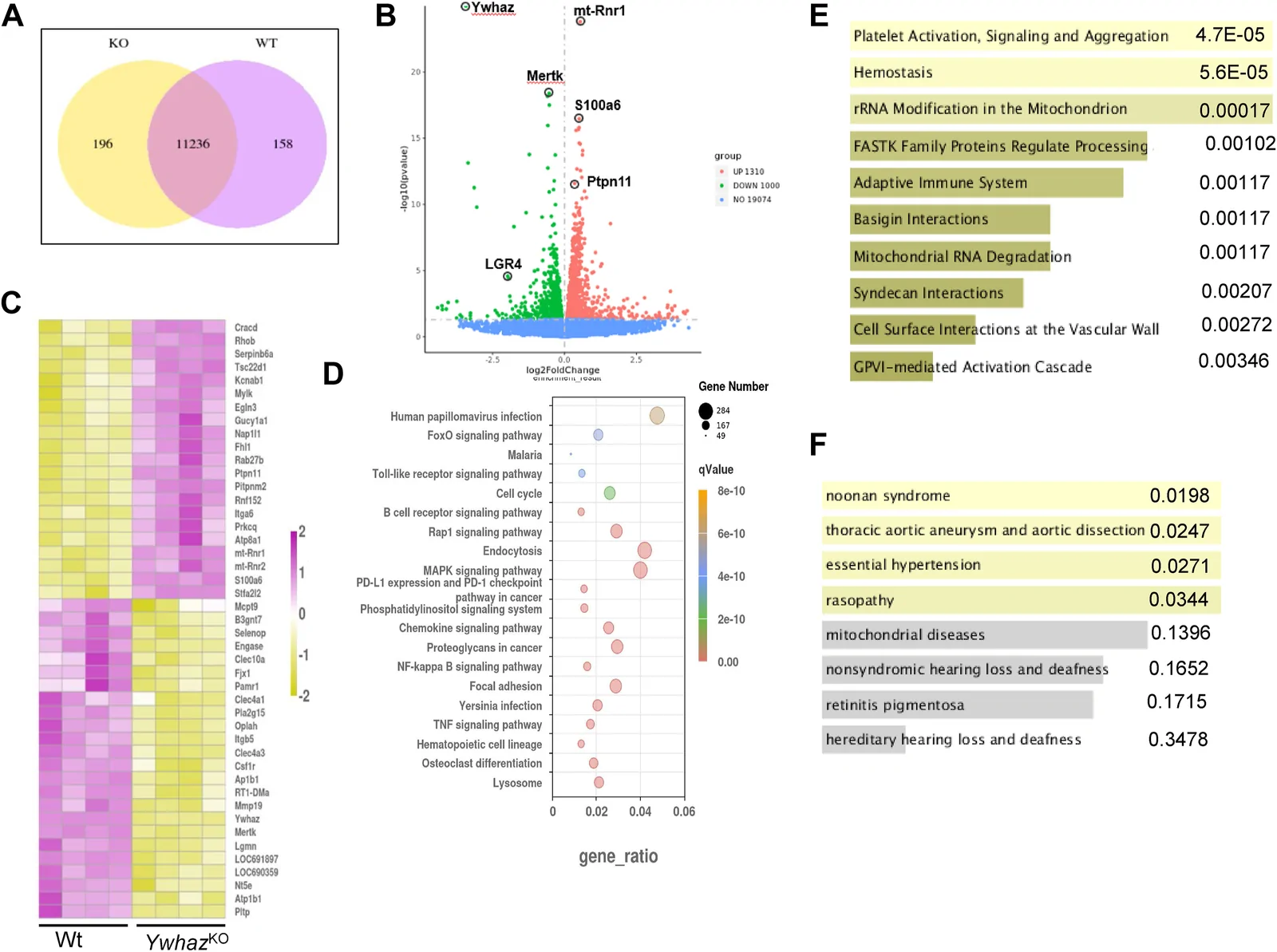
**14-3-3ζ suppresses inflammation and cellular adhesion at basal level.***A*, venn diagram showing the number of differentially expressed and overlapping genes of untreated WT and *Ywhaz*^KO^ rat BMDM. *B*, volcano plot represents the overall distribution of DEGs in untreated WT and *Ywhaz*^KO^ rat BMDM. *C*, heatmap analysis of *top* 50 DEGs (*p*-value ≤ 0.05, |log2(fold change) | >=1). *D*, KEGG analyses of selected differentially regulated pathways altered in *Ywhaz*^*KO*^ rat BMDM. *E*, the Reactome pathway analysis shows genes that are enriched in specific pathways. *F,* the *ClinVar* analysis shows the associations of affected genes to human diseases. The *p*-values of significance are listed for individual pathways shown in *E* and *F*. The results shown are the averages of 2 biological and 2 technical replicates. BMDM, bone marrow-derived macrophage.

### 14-3-3ζ suppresses RANKL-induced chemokine production

To define transcriptional responses regulated by 14-3-3ζ, we compared gene expression in WT and *Ywhaz*^KO^ BMDM at 8 h post-RANKL stimulation. To focus specifically on RANKL-dependent effects, genes differentially expressed at baseline between WT and *Ywhaz*^KO^ were excluded from further analysis. Venn diagram analysis identified 1326 genes uniquely expressed in RANKL-stimulated *Ywhaz*^*KO*^ BMDM; after removing basal differences, approximately 612 RANKL-induced genes were selectively influenced by 14-3-3ζ (Figs. 2*A* and S3*A*). Consistent with this, volcano plot analysis demonstrated robust transcriptional activation in *Ywhaz*^*KO*^ BMDM at 8 h, including significant upregulation of *Ltbp1*, *Cxcl3*, Ccl7 and *Ccl12* (Fig. 2*B*).

**Figure 2:**
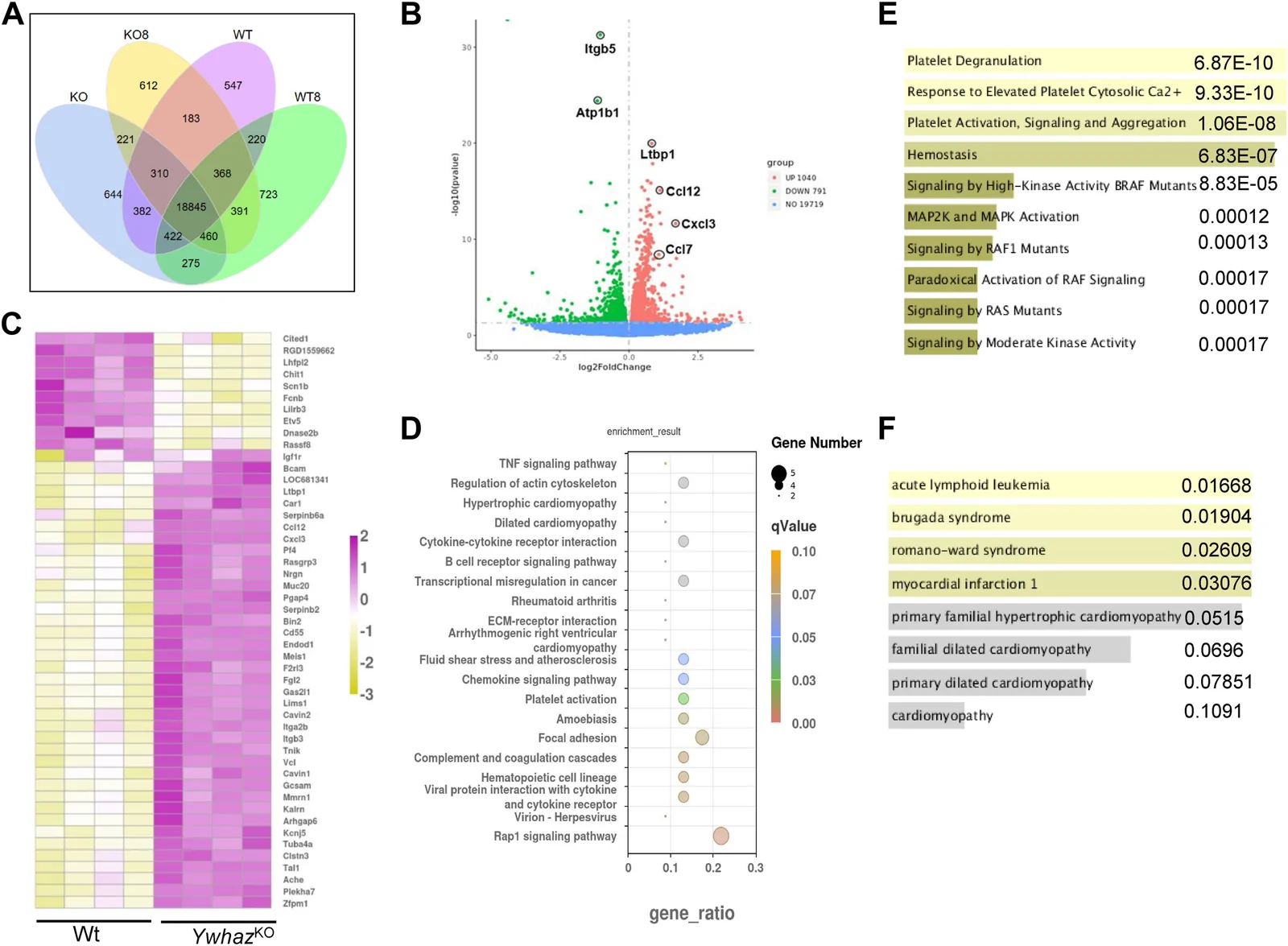
**14-3-3ζ suppresses RANKL-induced chemokine production.***A*, venn diagram showing the number of differentially expressed and overlapping genes in WT and *Ywhaz*^KO^ rat BMDM treated with M-CSF/RANKL for 8 h. *B*, volcano plot represents the overall distribution of DEGs in WT and *Ywhaz*^KO^ BMDM treated with M-CSF/RANKL for 8 h. *C*, heatmap analysis of *top* 50 DEGs (*p*-value ≤ 0.05, |log2(fold change)| >=1). *D*, KEGG analyses of selected differentially regulated pathways altered in *Ywhaz*^*KO*^ BMDM. *E*, reactome pathway analysis shows the genes that are enriched in specific pathways. *F*, the *ClinVar* analysis shows the associations of affected genes to human diseases. The *p*-values of significance are listed for individual pathways shown in *E* and *F*. The results shown are the averages of 2 biological and 2 technical replicates. BMDM, bone marrow-derived macrophage; M-CSF, macrophage colony-stimulating factor.

Heatmap analysis of the top 50 DEGs revealed increased expression of cytokine-associated genes (*Ccl12*, *Cxcl3*, *Igf1r*) and cytoskeletal or adhesion-related genes (*Itga2b*, *Itgb3*) in *Ywhaz*^KO^ macrophages, alongside reduced expression of genes linked to lymphoid leukemia (*Lilrb3*, *Etv5*) (Figs. 2*C* and S3*B*). Notably, *Cd55*, *Kcnj5*, and *Pgap4* were prominently upregulated in *Ywhaz*^KO^. While CD55 is a recognized regulator of osteoclast differentiation, the direct involvement of *Kcnj5* (a potassium ion channel), *Cavin* (a caveolae-associated protein), or *Pgap4* (a GPI-anchor remodeling enzyme) in osteoclastogenesis or RANKL signaling has not been previously reported (22). Enrichment and Reactome analyses indicated that early transcriptional changes predominantly affected Rap1 signaling and focal adhesion pathways (Figs. 2, *D* and *E* and S3, *C* and *D*), while *ClinVar* analysis linked altered ion channel genes to clinical conditions including Brugada syndrome, Romano-Ward syndrome, and myocardial infarction (Fig. 2*F*). Collectively, these findings demonstrate that loss of 14-3-3ζ amplifies early RANKL-induced transcriptional programs involving cytokine signaling, ion channels, and extracellular matrix interactions, positioning 14-3-3ζ as an early regulator of signaling events that shape osteoclast differentiation outcomes.

### Absence of 14-3-3ζ promotes lysosomal involvement upon RANKL stimulation

To investigate intermediate transcriptional changes during RANKL-induced osteoclast differentiation, we analyzed gene expression at 20 h post-stimulation. RNA-seq analysis identified 1346 uniquely expressed genes in *Ywhaz*^KO^ BMDM. After excluding basal genotype-specific differences observed in naïve *Ywhaz*^KO^ BMDM, 76 RANKL-induced transcripts were upregulated in *Ywhaz*^KO^ compared with 44 transcripts in WT cells (Figs. 3*A* and S4*A*). Volcano plot analysis revealed significant transcriptomic changes in *Ywhaz*^KO^ at 20 h, including pronounced upregulation of *Ccl7, Loxl2*, and *Ezr*, while downregulation of a subset of genes, such as *Apoe*, and *Tmem176b* (Fig. 3*B*). Heatmap analysis of the top 50 DEGs shows that most genes at this time point were downregulated, Apoe and Slc46a3 being prominent ones. While *Ezr*, *Loxl2*, and *Ccl7*, genes associated with actin remodeling, extracellular matrix cross-linking, and chemokine signaling, respectively, were significantly upregulated in *Ywhaz*^KO^ (Fig. 3*C*).

**Figure 3:**
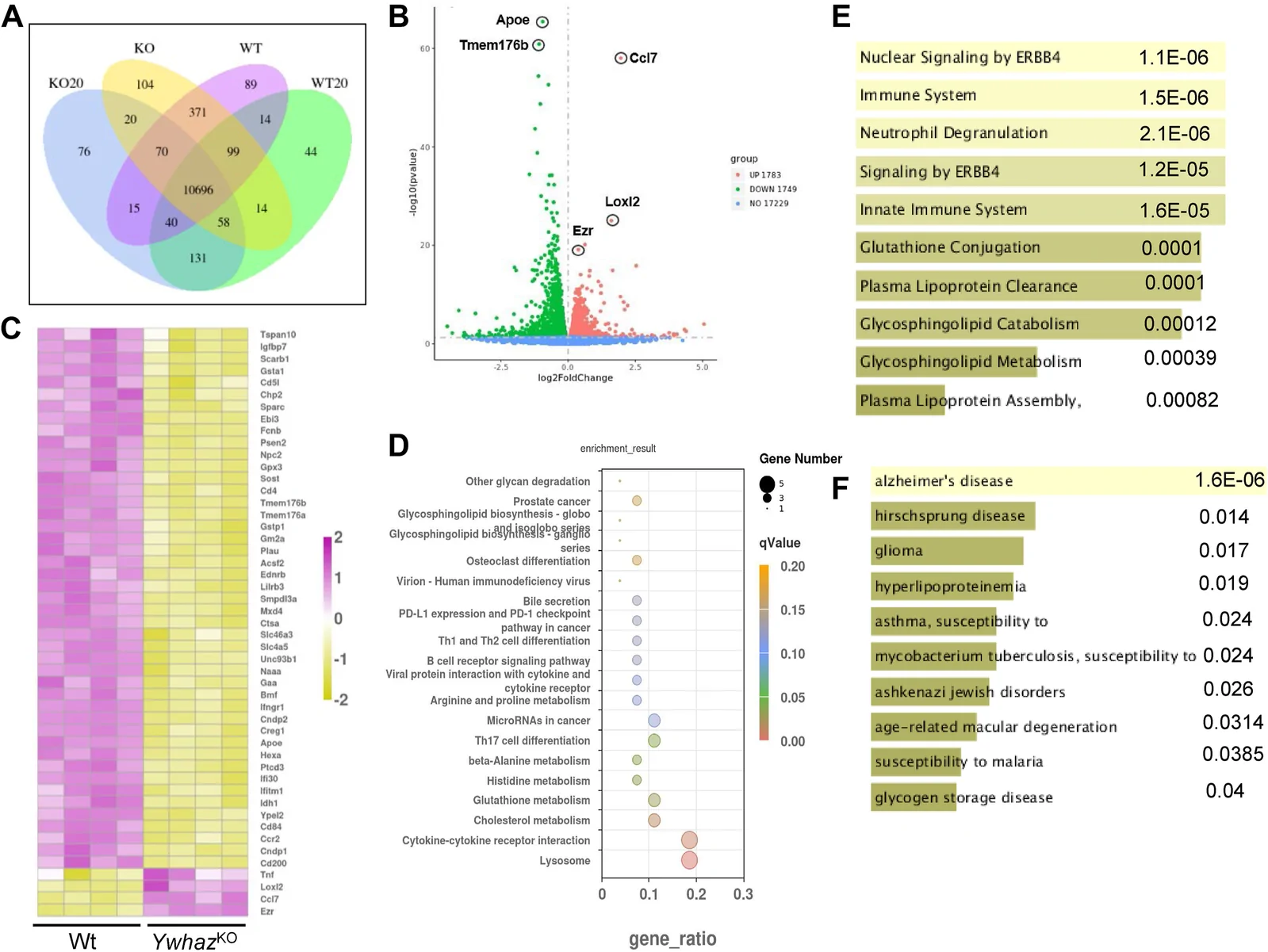
**14-3-3ζ regulates lysosome involvement in mid-time RANKL signaling.***A*, venn diagram showing the number of differentially expressed and overlapping genes in untreated WT and *Ywhaz*^KO^ rat BMDM treated with M-CSF/RANKL for 20 h. *B*, *volcano plot* represents the overall distribution of DEGs in WT and Y*whaz*^KO^ BMDM. *C*, heatmap analysis of *top* 50 DEGs (*p*-value ≤ 0.05, |log2(fold change) | >=1). *D*, KEGG analyses of selected differentially regulated pathways altered in *Ywhaz*^KO^ BMDM. *E*, reactome pathway analysis shows the genes that are enriched in specific pathways. *F*, the *ClinVar* analysis shows the associations of affected genes to human diseases. The *p*-values of significance are listed for individual pathways shown in *E* and *F*. The results shown are the averages of 2 biological and 2 technical replicates. BMDM, bone marrow-derived macrophage; M-CSF, macrophage colony-stimulating factor.

Pathway enrichment and Reactome analyses revealed significant upregulation of genes involved in lysosomal pathways and cytokine-cytokine receptor interactions in RANKL-stimulated *Ywhaz*^KO^ BMDM at 20 h (Fig. 3*D*). These transcriptional changes were linked to altered immune cell signaling, prominently involving the HER kinase pathway, as well as cellular redox processes. *ClinVar* analysis identified Alzheimer’s disease as the most significantly associated condition (Fig. 3, *E* and *F*). Cluster analysis further identified coordinated regulation of *ApoE*, *PSEN2*, *SCARB1*, and *GSTP1*, genes implicated in determining disease-associated phenotypes (Fig. S4*B*). Consistently, gene ontology analysis indicated significant upregulation of pathways related to peptidase activity, ion channel activity, and lysosomal and vesicular processes in *Ywhaz*^KO^ (Fig. S4, *C* and *D*). Given that enhanced lysosomal function is known to support osteoclast activity, these results indicate that 14-3-3ζ suppresses RANKL-stimulated lysosomal engagement at the mid-point of osteoclast differentiation (23, 24). Together with the early amplification of inflammatory and chemokine programs, these mid-point changes indicate that loss of 14-3-3ζ promotes engagement of lysosomal and vesicular pathways, setting the stage for extensive extracellular matrix remodeling and heightened osteoclast activity at later stages of RANKL signaling.

### 14-3-3ζ modulates RANKL-induced transcripts regulating extracellular matrix remodeling

The transcriptional responses to RANKL at 40 h post-stimulation were markedly amplified in *Ywhaz*^KO^ BMDM compared with WT cells. RNA-seq analysis at 40 h revealed 1416 unique RANKL-responsive transcripts in *Ywhaz*^KO^, even after accounting for basal genotype-dependent differences. After correcting for basal genotype-dependent differences, 93 transcripts were identified as uniquely regulated by RANKL signaling in the absence of 14-3-3ζ (Figs. 4*A* and S5*A*). The volcano plot analysis revealed 2180 differentially expressed transcripts at 40 h following RANKL stimulation of *Ywhaz*^KO.^ The prominent ones include upregulation of *Serpin6a* and downregulation of MHC class II (*RT1-Da* and *RT1-Bb*) (Fig. 4*B*). Heatmap analysis of the top 50 DEGs demonstrated significant upregulation of *Fos*, *Ccl7*, *Melk*, and *Vim* in *Ywhaz*^KO^ relative to WT BMDM (Fig. 4*C*). Network clustering analysis of top DEG suggests *Atp1b1*, encoding the β1 subunit of the Na^+^/K^+^-ATPase plasma membrane complex, is an effector of 14-3-3ζ′s role in the RANKL-induced transcriptional network (Fig. S5*B*).

**Figure 4:**
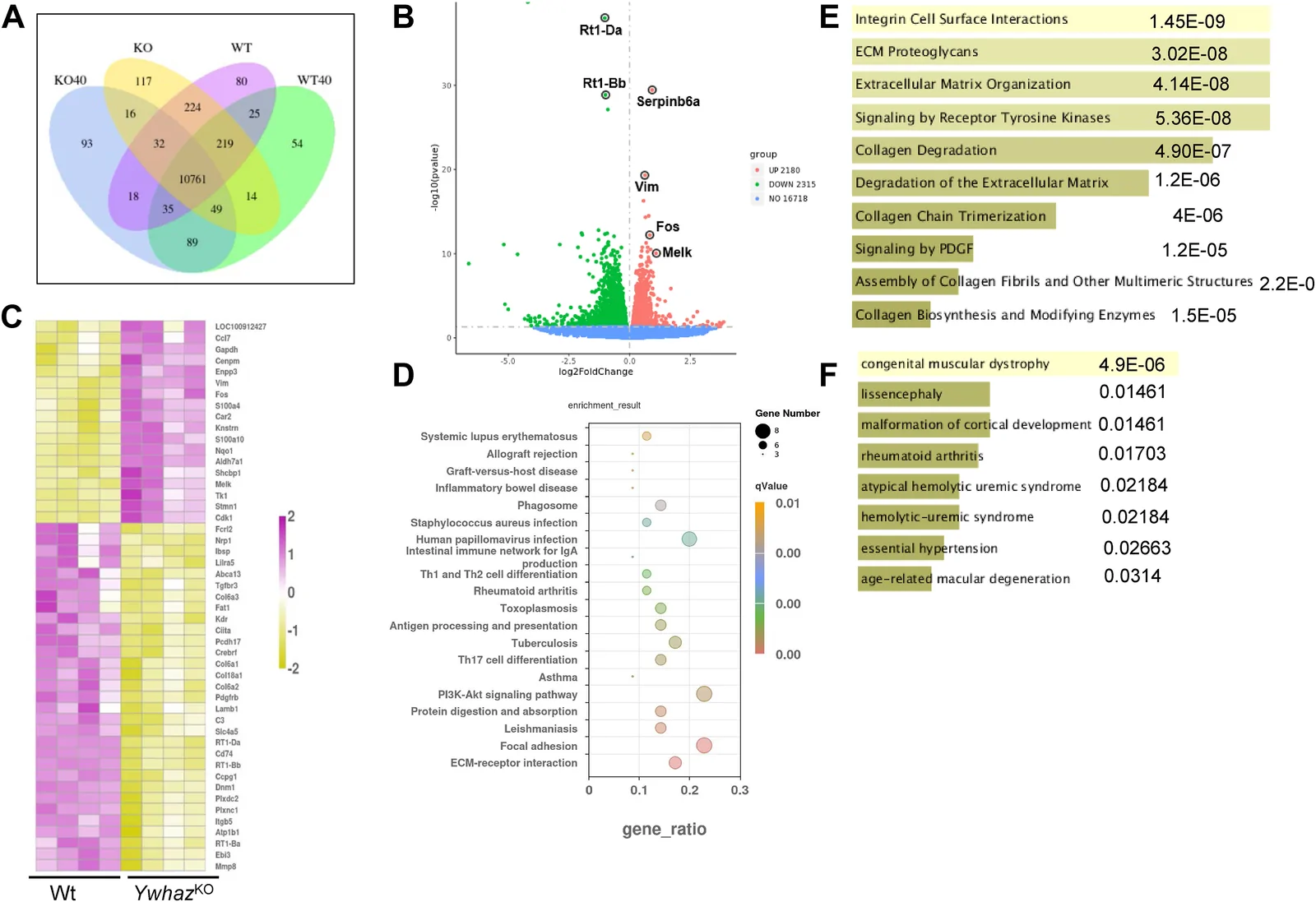
**14-3-3ζ regulates RANKL-induced extracellular matrix remodeling at late time points.***A*, venn diagram showing the number of differentially expressed and overlapping genes in WT and Y*whaz*^*KO*^ rat BMDM treated with M-CSF/RANKL for 40 h. *B*, *volcano plot* represents the overall distribution of DEGs in WT and Y*whaz*^KO^ BMDM treated with M-CSF/RANKL for 40 h. *C*, heatmap analysis of *top* 50 DEGs (*p*-value ≤ 0.05, |log2(fold change) | >=1). *D*, KEGG analyses of selected differentially regulated pathways altered in *Ywhaz*^*KO*^ rat BMDM. *E*, reactome pathway analysis shows the genes that are enriched in specific pathways. *F*, the *ClinVar* analysis shows the associations of affected genes to human diseases. The *p*-values of significance are listed for individual pathways shown in *E* and *F*. BMDM, bone marrow-derived macrophage; M-CSF, macrophage colony-stimulating factor.

Pathway enrichment and Reactome analyses revealed significant alterations in focal adhesion, extracellular matrix-receptor interaction, and PI3K-Akt signaling pathways at this late time point (Fig. 4, *D* and *E*). Gene ontology analysis further indicated that collagen-associated processes and VEGFR signaling activities were among the most significantly affected biological functions regulated by 14-3-3ζ (Fig. S5, *C* and *D*). *ClinVar* analysis linked the dysregulated gene set to human diseases associated with aberrant extracellular matrix remodeling, including congenital muscular dystrophy, rheumatoid arthritis, and age-related macular degeneration (Fig. 4*F*).Collectively, these results indicate that prolonged RANKL stimulation in the absence of 14-3-3ζ drives extensive transcriptional remodeling of extracellular matrix-related pathways. Many of the affected genes have established roles in osteoclastogenesis and bone loss observed in myeloma, rheumatoid arthritis, *etc.*, supporting a role for 14-3-3ζ in restraining pathological extracellular matrix remodeling at late stages of osteoclast differentiation (5, 6). We examined genes involved in ECM pathways and found *Rac2* expression to be significantly increased in the absence of 14-3-3ζ and further stimulated by RANKL treatment (Fig. S5*E*). Importantly, *Rac2* has been shown to promote osteoclast survival, migration, and actin reorganization, thereby increasing osteoclast activity (25).

### 14-3-3ζ regulates lysosome-associated osteoclastic gene during RANKL signaling

To identify osteoclast-specific gene programs regulated by 14-3-3ζ, we performed a focused analysis of osteoclast differentiation signatures in *Ywhaz*^KO^ cells. Heatmap visualization of the top DEGs revealed robust upregulation of well-established osteoclast markers, including *Tnfrsf11a* (RANK), *Acp5* (TRAP5), and *Ctsk*, in *Ywhaz*^KO^ compared with WT macrophages (Fig. 5, *A* and *B*). In addition to these canonical markers, several previously unrecognized 14-3-3ζ-regulated genes were identified, including *Ifngr2*, *Ctsz*, *Fos*, *Calcr*, *Atp6v0c*, and *Car2*. Notably, *Atp6v0c*, a subunit of the vacuolar H^+^-ATPase, is essential for lysosomal acidification; *Car2* promotes extracellular matrix acidification and cell-cell fusion; and *Calcr* regulates calcium homeostasis during osteoclast differentiation (26, 27, 28). Given the strong enrichment of lysosome-associated genes, we next examined the impact of 14-3-3ζ on RANKL-induced lysosomal pathways. Analysis of the top 25 DEGs revealed that lysosomal genes were largely unchanged or modestly reduced under basal conditions in *Ywhaz*^KO^ cells. However, RANKL stimulation markedly induced expression of *Laptm4a*, *Clta*, *Ctsz*, *Lamp1*, and *Atp6v0c* specifically in the absence of 14-3-3ζ (Fig. 5*C*), indicating exaggerated activation of lysosome-associated gene programs.

**Figure 5:**
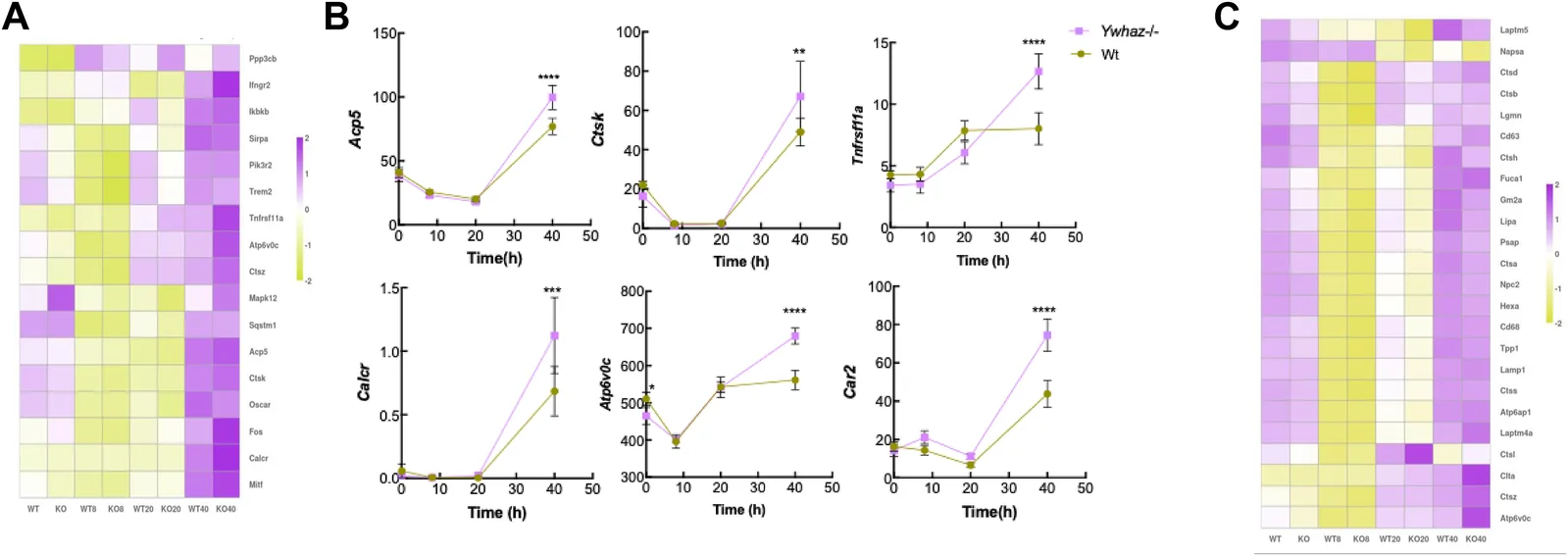
**14-3-3ζ targeted RANKL-induced osteoclastic genes.***A*, heatmap depicting the osteoclast-specific genes differentially regulated in WT and *Ywhaz*^KO^ rat BMDM treated with or without M-CSF/RANKL for the indicated time points. *B*, the FPKM values obtained for genes such as *Acp5, Ctsk, Tnfrs11a, Calcr, Atp6v0c,* and *Car2* were plotted for the indicated time points. The *asterisks∗∗, ∗∗∗,* and ∗∗∗∗ show *p*-value of <0.01, 0.005, and 0.001, respectively. *C*, Heatmap shows lysosome-associated genes affected by RANKL in WT or *Ywahz*^KO^ cells. BMDM, bone marrow-derived macrophage; M-CSF, macrophage colony-stimulating factor.

To functionally validate these findings, lysosomal content was assessed using LysoBrite dye and flow cytometry. The zombie dye was used to exclude dead cells and measure fluorescence in the live cells. The *Ywhaz*^KO^ RAW cells exhibited significantly increased fluorescence, as shown by histogram and mean fluorescence intensity (MFI) per live cell compared with WT, following 40 h of RANKL stimulation (Fig. 6*A*). A similar increase in LysoBrite histogram and MFI per live cell was observed in RANKL-treated *Ywhaz*^KO^ BMDM relative to WT (Fig. 6*B*). We then examined expression of specific lysosomal proteins, LAMP1 and CTSZ, on the RANKL-stimulated BMDM. Both LAMP1 and CTSZ staining were stronger in *Ywhaz*^KO^ cells than in WT (Figs. 6, *C* and *D* and S6, *A*–*D*). Quantification of fluorescence intensity normalized per nucleus (including non-osteoclasts) demonstrated significantly elevated LAMP1 and a marginal increase in CTSZ signals in *Ywhaz*^KO^ (Fig. S6, *E* and *F*). However, when immunostaining was quantified specifically for osteoclasts, significant increases in both LAMP1 and CTSZ were observed in *Ywhaz*^KO^ (Fig. 6, *E* and *F*). Together, these data indicate that loss of 14-3-3ζ amplifies RANKL-induced lysosomal gene expression and activity.

**Figure 6:**
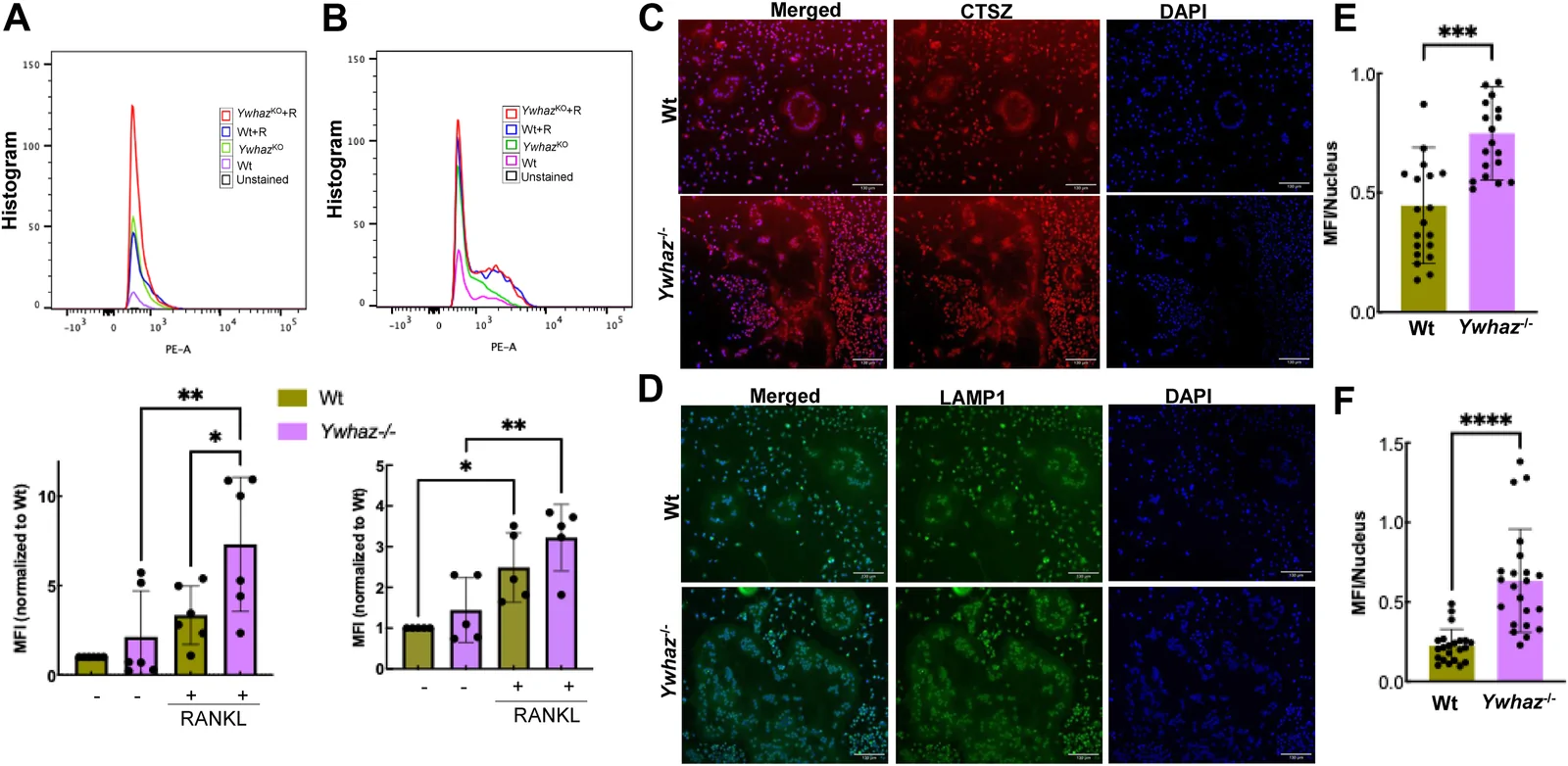
**14-3-3ζ suppresses lysosomal activity in the RANKL signaling.***A*, overlays of fluorescence spectra of WT and *Ywhaz*^KO^ RAW cells treated with M-CSF/RANKL (40 h) and LysoBrite*Red* (30 min) followed by flow cytometry measurement, are shown. Representative histogram and mean fluorescence intensity (MFI) quantification from six independent repeats (n = 6) are shown. *B*, representative overlays of fluorescence spectra and quantification of MFI of five biological repeats (n = 5) with WT and *Ywhaz*^KO^ BMDM treated with M-CSF/RANKL (40 h) and LysoBriteTMRed (30 min) followed by flow cytometry are shown. *C*, primary BMDM from WT and *Ywhaz*^KO^ animals were treated with M-CSF/RANKL for 4 days followed by anti-Cathepsin Z (CTSZ) staining and imaged by ECHO fluorescence microscopy. Representative images are shown. *D*, primary BMDMs from WT and *Ywhaz*^KO^ animals were treated with M-CSF/RANKL for 4 days followed by anti-Lamp1 staining and imaged by ECHO fluorescence microscopy. Representative images are shown. *E*, the osteoclast fluorescence intensity of CTSZ from four biological replicates and 5 microscopic fields from each study was quantified and normalized to DAPI using ImageJ (n = 4). *F*, the osteoclast fluorescence intensity of LAMP1 from four biological replicates and 5 microscopic fields from each study was quantified and normalized to DAPI using ImageJ (n = 4). The scale bars are shown. An ordinary one-way ANOVA was performed for *panelsA* and *B* and Welch's *t* test was used for panels *E* and *F*. The *asterisks ∗*, *∗∗*, *∗∗∗*, *and ∗∗∗∗* indicate *p*-value <0.05, 0.01, 0.005, and 0.001, respectively. BMDM, bone marrow-derived macrophage; M-CSF, macrophage colony-stimulating factor.

### 14-3-3ζ negatively regulates RANKL-induced CCL7 expression

The transcriptomic analysis indicated that multiple inflammatory cytokines are regulated by 14-3-3ζ during RANKL signaling. To validate, we performed independent experiments. The time-course analysis of RANKL-stimulated BMDM revealed a significantly greater induction of *Ccl7* in *Ywhaz*^KO^ compared with WT macrophages (Fig. 7*A*). Notably, CCL7 has been associated with osteoclast recruitment, osteomyelitis, and osteoporosis in post-menopausal women (29). A similar trend was observed for *Ccl12*, which was also significantly increased in *Ywhaz*^KO^ cells throughout the RANKL stimulation time course (Fig. S7*A*).

**Figure 7:**
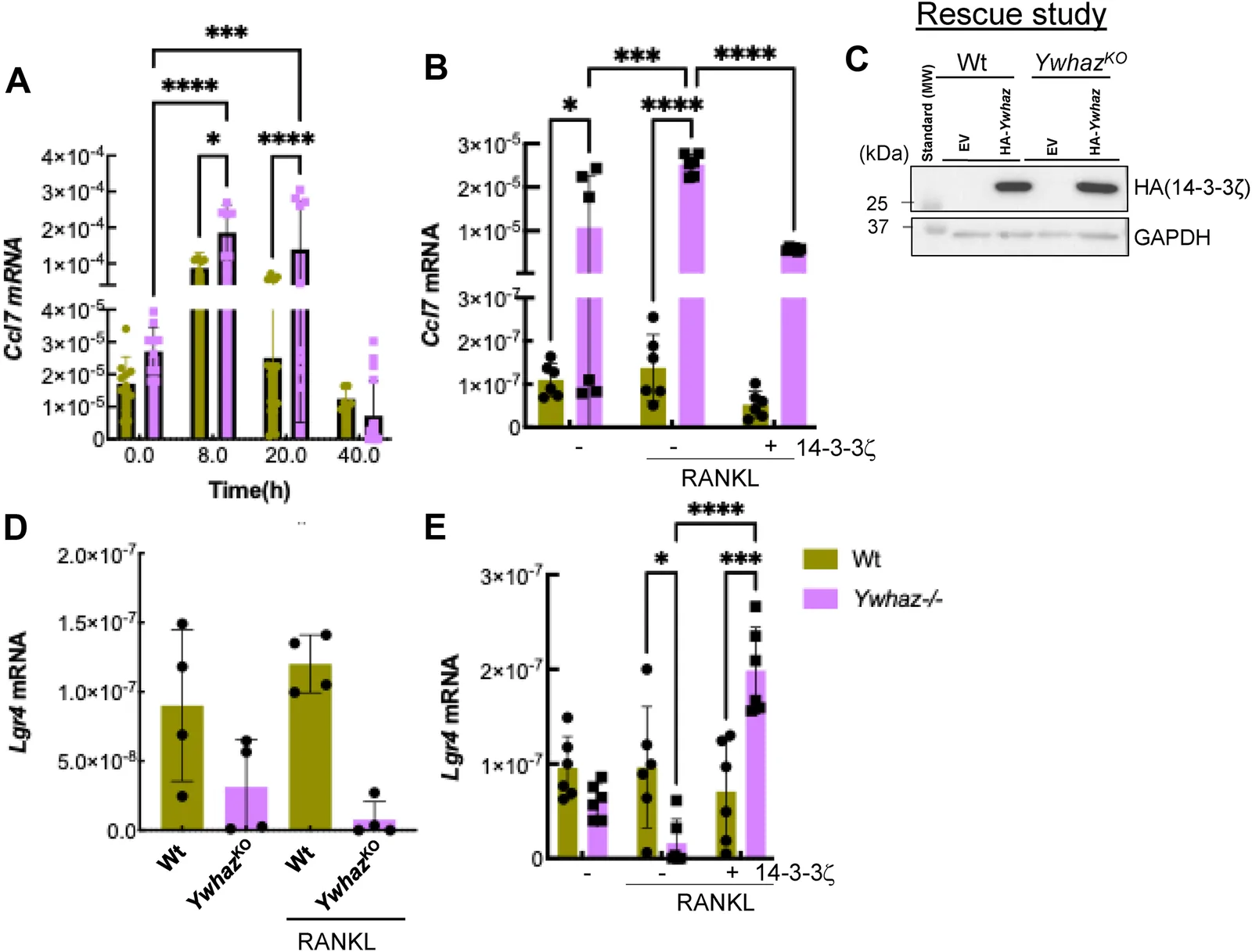
**14-3-3ζ differentially regulates Ccl7 and Lgr4 in the RANKL singling.***A*, time course of *Ccl7* transcript in WT and *Ywhaz*^KO^ rat BMDM treated with M-CSF/RANKL at the indicated time points is shown (n = 3). *B*, the mRNA levels of *Ccl7* in WT and *Ywhaz*^KO^ RAW cells overexpressing HA-14-3-3ζ followed by M-CSF/RANKL treatment for 20 h, and qRT-PCR analysis, are shown (n = 3). *C*, expression of HA-14-3-3ζ is shown by immunoblot using anti-HA antibody. GAPDH is shown as a loading control. Molecular weights are shown on the *left* of the immunoblot panels. Original blots are shown in the supplementary data. *D*, *Lgr4* transcript in WT and *Ywhaz*^KO^ rat BMDM treated with M-CSF/RANKL at 20 h post-treatment is shown (n = 3). *E*, the mRNA levels of *Lgr4* in WT and *Ywhaz*^KO^ RAW cells overexpressing HA-14-3-3ζ followed by M-CSF/RANKL treatment for 20 h and qRT-PCR analysis are shown (n = 3). The expression levels of the mRNAs were normalized to 18S rRNA. HA-14-3-3ζ expression is shown in the *panel C*. The data represented as mean ± SD. The two-way ANOVA test was performed for *panelsA, B, D* and *E*. *Asterisks ∗*, *∗∗, ∗∗∗ and ∗∗∗∗* show *p*-values of <0.05, <0.01, 0.005 and 0.001, respectively. BMDM, bone marrow-derived macrophage; M-CSF, macrophage colony-stimulating factor.

To directly assess whether 14-3-3ζ regulates *Ccl7* expression, we transiently transfected *Ywhaz*^KO^ and WT Raw cells with HA-tagged 14-3-3ζ. Robust 14-3-3ζ expression was confirmed in both cell types (Figs. 7*C* and S7*B*). Rescue of 14-3-3ζ in *Ywhaz*^KO^ resulted in a marked suppression of RANKL-induced *Ccl7* expression; in comparison, a milder effect was observed when overexpressed in the WT cells (Fig. 7*B*). This confirms that 14-3-3ζ negatively regulates *Ccl7* during RANKL signaling. In addition to inflammatory cytokines, *Ywhaz*^KO^ exhibited elevated *Slc6a4* expression at baseline and at 20 h after RANKL stimulation. Because *Slc6a4* encodes a serotonin transporter, we examined whether extracellular serotonin levels were altered in 14-3-3ζ deficiency. Measurement of serotonin concentrations in conditioned media from RANKL-stimulated BMDM revealed no significant differences between WT and *Ywhaz*^KO^ cells (Fig. S7*C*), indicating that 14-3-3ζ does not appreciably affect extracellular serotonin levels under these conditions.

### 14-3-3ζ constrains RANKL signaling by regulating SHP-2 and LGR4

While the results above established a role for 14-3-3ζ in suppressing RANKL-induced inflammatory cytokine output, we next asked how 14-3-3ζ modulates the amplitude of RANKL signaling during osteoclast differentiation. To this end, we next investigated whether 14-3-3ζ influences the expression of negative regulators of RANKL-induced osteoclastogenesis. Our data show that several negative regulators of RANKL signaling, including *Lgr4*, *Wdfy3*, and *Apoe*, were downregulated in *Ywhaz*^KO^ (Figs. 3 and S7, *D* and *E*) (30, 31). LGR4 is a G-protein-coupled receptor that antagonizes RANKL signaling by competing with RANK for ligand binding (32). The *Ywhaz*^KO^ cells exhibited reduced basal LGR4 expression, which became undetectable following RANKL stimulation, while WT BMDMs maintained *Lgr4* expression post-RANKL stimulation (Fig. 7*D*). Importantly, transient rescue of 14-3-3ζ restored LGR4 levels in *Ywhaz*^KO^ cells (Figs. 7*E* and S7*B*), demonstrating that 14-3-3ζ positively regulates LGR4.

In contrast to Lgr4, *Ywhaz*^KO^ macrophages exhibited significantly increased expression of *Ptpn11* (Figs. 1, *B* and *C* and S7*F*). *Ptpn11* (SHP-2) is a dual-specificity phosphatase known to promote osteoclast precursor fusion and differentiation (17). Increased SHP-2 protein levels were confirmed in *Ywhaz*^KO^ RAW cells (Figs. 8*A* and S8*A*). To determine whether 14-3-3ζ directly regulates SHP-2 expression, we transiently overexpressed FLAG-tagged 14-3-3ζ in both WT and *Ywhaz*^KO^ RAW cells. Elevated 14-3-3ζ expression led to a marked reduction in SHP-2 protein levels in both genotypes (Figs. 8, *B* and *C* and S8, *B* and *C*), indicating that 14-3-3ζ negatively regulates SHP-2 protein levels.

**Figure 8:**
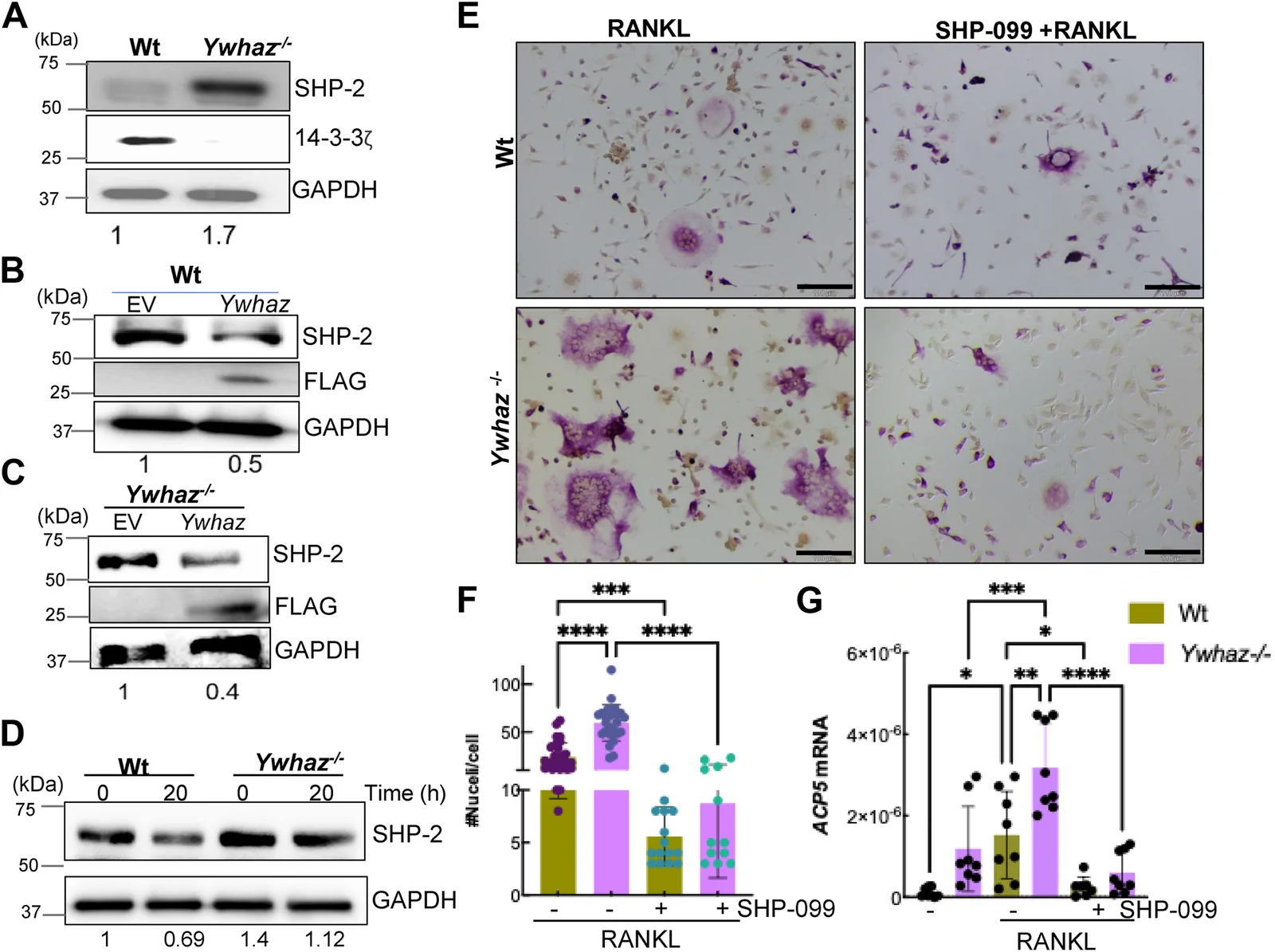
**14-3-3ζ suppresses SHP-2 in the RANKL singling.***A*, the SHP-2 protein expression level from WT and *Ywhaz*^KO^ RAW cells was analyzed by immunoblot (n = 3). *B* and *C*, WT and *Ywhaz*^KO^ RAW cells transfected with either empty vector or FLAG-*Ywhaz,* and the effect on SHP-2 protein level was analyzed by immunoblot (n = 3). Expression of FLAG indicates succesful transfection and expression of 14-3-3ζ. *D*, effect of RANKL treatment on SHP-2 levels in WT and *Ywhaz*^KO^ RAW was analyzed by immunoblot (n = 3). For *panelsA* and *D*, representative blots are shown. Band intensities in comparison to GAPDH were quantified and are listed under the lane. Original blots are shown in the supplementary figure (Fig. S8). *E*, the effect of SHP-2 inhibitor (SHP-099) on the osteoclast differentiation assay in WT and *Ywhaz*^KO^ BMDM is shown. BMDMs were co-treated with or without SHP-2 inhibitor (10 μM) during the 4 days of M-CSF and RANKL followed by TRAP staining. Representative images with the scale bar represents 110 μm are shown. *F*, the number of nuclei per TRAP+ osteoclast (>3 nuclei) in RANKL-treated WT and *Ywhaz*^KO^ BMDM from four independent repeats and 4 microscopic fields per experiment. *G*, the effect of SHP-099 on the RANKL-treated WT and *Ywhaz*^KO^ RAW cells (n = 4) on *Acp5* induction was measured by qRT-PCR. The expression levels of the mRNAs were normalized to 18S rRNA. An ordinary one-way ANOVA test was performed for *F* and *G*. The *asterisks ∗, ∗∗,∗∗∗, and ∗∗∗∗* indicate *p*-value of <0.05, 0.01, 0.005, and 0.001, respectively. BMDM, bone marrow-derived macrophage; M-CSF, macrophage colony-stimulating factor.

Because the *Ptpn11* transcript (Fig. S7*F*) was reduced by RANKL stimulation, we next examined how 14-3-3ζ influences SHP-2 protein levels following RANKL exposure. Treatment with RANKL decreased SHP-2 protein levels in WT; however, SHP-2 levels remained consistently higher in *Ywhaz*^KO^, as shown by immunoblot and quantifications shown underneath (Figs. 8*D* and S8*D*). To determine whether elevated SHP--2 functionally contributes to enhanced osteoclast differentiation in *Ywhaz*^KO^, we treated cells with SHP-099, a highly selective allosteric SHP-2 inhibitor (33). Inhibition of SHP-2 significantly reduced RANKL-induced TRAP+ osteoclasts in the *Ywhaz*^KO^. Quantification of the number of nuclei per osteoclast showed a significant decrease in the presence of SHP-099 (Figs. 8, *E* and *F* and S8*E*). Similarly, *Acp5* expression in SHP-099-treated *Ywhaz*^KO^ was substantially reduced, indicating that increased SHP-2 activity contributes to enhanced osteoclastogenic signaling downstream of 14-3-3ζ (Fig. 8*G*). Together, these findings identify SHP-2 as a key downstream effector through which 14-3-3ζ constrains RANKL signaling.

Overall, our results demonstrate that 14-3-3ζ functions as a critical brake on RANKL-induced osteoclast activation by simultaneously suppressing positive regulators such as SHP-2 and supporting inhibitory pathways, such as LGR4. Through this coordinated regulation, 14-3-3ζ limits osteoclast-specific gene expression and inflammatory cytokine production, including *CCL7*, thereby restraining excessive osteoclastogenesis (Fig. 9).

**Figure 9:**
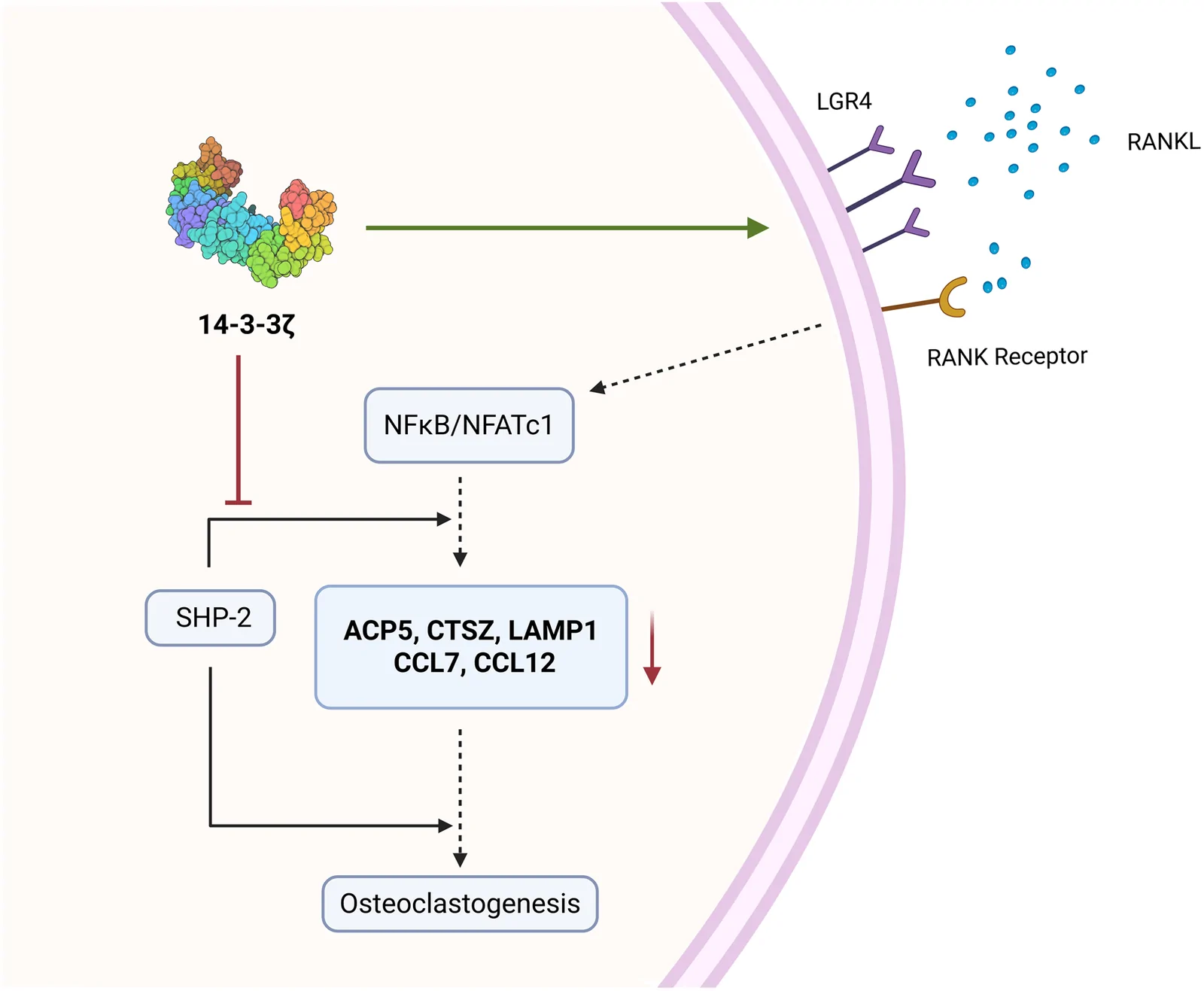
**Multi-level transcriptional regulation of RANKL signaling by 14-3-3ζ.** This study identifies 14-3-3ζ as a critical transcriptional checkpoint that limits RANKL-induced osteoclastogenesis. Using a time-course transcriptomics study, we show that 14-3-3ζ suppresses canonical osteoclast gene expression while regulating LGR4 and SHP-2 to control RANKL signaling and osteoclast differentiation. Pharmacologic inhibition of SHP-2 abolishes osteoclast differentiation in the absence of 14-3-3ζ, positioning SHP-2 downstream of this regulatory axis. These findings uncover a previously unrecognized layer of control in RANKL signaling and establish 14-3-3ζ as a key modulator of osteoclast differentiation.

## Discussion

In this study, we identify 14-3-3ζ as a critical regulator of osteoclast differentiation by restraining RANKL-dependent signaling. Building on our prior finding that 14-3-3ζ suppresses RANKL-induced osteoclastogenesis through post-translational mechanisms (19), we used unbiased transcriptomic profiling to show that loss of 14-3-3ζ broadly amplifies RANKL-induced gene expression without substantially altering pathway identity. As anticipated, RANKL treatment of WT and *Ywhaz*^KO^ BMDMs for 40 h showed temporal changes in transcripts (Fig. S9A-B). However, in-depth analysis showed significant changes in the top 200 DEGs, thereby triggering an in-depth analysis of how 14-3-3ζ affects the transcriptional program in response to RANKL stimulation. Time-course analysis revealed that 14-3-3ζ modulates immune and cytoskeletal programs throughout osteoclastogenesis, with early effects on chemokine signaling (8 h) and later effects on lysosomal activity, extracellular matrix remodeling (*Rac2*), and intracellular trafficking (20–40 h). The largely preserved qualitative transcriptional landscape in *Ywhaz*^KO^ cells suggests that 14-3-3ζ primarily limits signaling amplitude rather than pathway selection. Consistent with this model, we identified that 14-3-3ζ regulates both positive and negative mediators of RANKL signaling, including suppression of *Ptpn11* (SHP-2), a known promoter of osteoclast differentiation (17), and induction of inhibitory regulators such as *Lgr4, Apoe,* and *Wdfy3* (30, 32). Together, these findings position 14-3-3ζ as a signaling rheostat that constrains osteoclastogenesis by coordinately targeting multiple regulatory nodes to dampen RANKL signaling output.

Prior work has established that gene induction in preosteoclasts dictates osteoclast fate, with RANKL/M-CSF-driven differentiation proceeding through sequential transcriptional programs involving receptors, chemokines, and signaling molecules (18). Consistent with this framework, we observed a marked increase in RANKL-induced transcription factors, including *c*-*Fos*, *Ikbkb*, and *Mitf1*, in *Ywhaz*^KO^ cells. Elevated *Ikbkb* expression in *Ywhaz*^KO^ likely contributes to enhanced NF-κB activity and increased inflammatory gene expression, including cytokines. Notably, *Mitf1* is a key regulator of terminal osteoclast differentiation, and its transcriptional activity has been reported to be regulated by 14-3-3ζ (34). Our data demonstrate that 14-3-3ζ suppresses RANKL-induced *Mitf1* expression, implicating this scaffold protein in transcriptional control during early osteoclastogenesis. In parallel, loss of 14-3-3ζ increased expression of chemokines such as *Ccl7*, *Ccl12*, and *Cxcl3*, all of which have established roles in osteoclast recruitment and bone loss. It is reported that CCL12 promotes RANKL expression and contributes to trabecular bone loss, whereas CCL7 functions as a chemotactic factor that enhances osteoclast accumulation at resorption sites (35, 36). Importantly, pharmacologic targeting of CCL7 using Bindarit has been shown to preserve bone mass (29). Our results show that changes in 14-3-3ζ level negatively regulate RANKL-induced CCL7 induction. Together, these findings indicate that 14-3-3ζ restrains early RANKL-driven transcriptional and chemokine programs, in addition to previously published role (targeting TRAF6) proximal to the receptor complex (19).

Consistent with these molecular findings, we previously demonstrated that *Ywhaz*^KO^ Lewis rats show significantly reduced trabecular bone volume and bone mineral density in models of inflammatory arthritis (7). Inflammatory bone loss is driven by a cytokine network that includes RANKL, IL-17A, and TNF-α, all potent inducers of osteoclastogenesis (37, 38). Although therapeutic inhibition of these pathways can preserve bone integrity, such approaches are associated with increased infection risk and other adverse effects, such as atypical fractures in the case of Denosumab, underscoring the need for alternative regulatory targets (39, 40, 41). In this context, emerging strategies targeting lysosomal and autophagy pathways have shown promise in autoimmune disease models (42). Increased expression of cathepsins and enhanced lysosomal biogenesis are closely linked to both autoimmune and bone pathologies (43, 44). Several new targets, including sugar transporters and lysosome-mitochondria crosstalk, have gained attention for their role in regulating osteoclast activity (45, 46). Preliminary evaluation of one such sugar transporter, *Slc37a2*, in our RNA-seq data also showed increased levels in the RANKL-stimulated *Ywhaz*^KO^ (Fig. S10), though further validation is needed. We believe that the elevated expression of lysosomal markers such as LAMP-1 and CTSZ observed in *Ywhaz*^KO^ macrophages is likely a consequence of amplified RANKL signaling; however, a direct role for 14-3-3ζ in regulating lysosomal biogenesis *via* MITF-dependent mechanisms cannot be excluded (47).

Our results indicate that, in addition to its previously published role in promoting TRAF6 degradation and interrupting proximal signal transduction, 14-3-3ζ regulates RANKL signaling by targeting multiple downstream regulatory nodes. SHP-2 (*PTPN11*), a dual-specificity phosphatase that is reported to promote cell fusion during osteoclastogenesis, was suppressed at basal levels by 14-3-3ζ, and its RANKL-induced degradation was enhanced in the presence of 14-3-3ζ. It is important to note that several mutations in *PTPN11* are associated with Noonan syndrome in humans (48). Although the precise mechanisms governing SHP-2 stability during RANKL signaling remain to be defined, these findings identify SHP-2 as a functionally relevant target of 14-3-3ζ-mediated restraint. In contrast, 14-3-3ζ promoted expression of *Lgr4*, which inhibits RANKL signaling, both at baseline and following RANKL stimulation. This role can impact the availability of RANKL to signal through RANK. Together, these data support a model in which 14-3-3ζ exerts coordinated transcriptional control over both positive and negative modulators, thereby functioning as an intrinsic brake that limits excessive RANKL signaling. While the temporal hierarchy of these regulatory events requires further investigation, this transcriptional restraint complements the previously described post-translational regulation of TRAF6, suggesting 14-3-3ζ has dual regulatory activity in RANKL signaling to limit osteoclast differentiation.

Collectively, these findings underscore a broader anti-inflammatory and bone-protective role for 14-3-3ζ, whose dysregulation has been linked to multiple pathological states, including rheumatoid arthritis and aging (49, 50), and highlight its potential as a therapeutic target to attenuate osteoclast-driven bone loss without the systemic liabilities of cytokine blockade.

## Experimental procedures

### Chemicals

All common chemicals were purchased from Fisher Scientific Inc unless stated otherwise. Rat and mouse M-CSF and RANKL were obtained from R&D Systems. The LAMP-1 antibody was purchased from Santa Cruz Biotechnology Inc. The antibodies (Cathepsin Z, SHP-2) were purchased from Cell Signaling Technology. The RNA isolation kit was purchased from Qiagen, and the RIN assessment kit was from Agilent Technologies Inc. All primers were synthesized by Integrated DNA Technologies (IDT), Inc.

### Cell culture

The RAW 264.7 cells were purchased from ATCC and maintained in Dulbecco's Modified Eagle's Medium (DMEM) supplemented with 10% FBS, penicillin, and streptomycin at 37 °C in a 5% CO_2_ incubator. The cells were pretreated with M-CSF (50 ng/ml) for 30 min, followed by mouse RANKL (50 ng/ml) treatment for the time as indicated later (51). The 14-3-3ζ KO RAW cells were generated using the CRISPR-Cas9 method as described before (52). Henceforth, the CRISPR-control plasmid-transfected cells are designated WT, and the 14-3-3ζ KO cells are designated *Ywhaz*^KO^. For overexpression or rescue, cells were transfected with FLAG/HA-14-3-3ζ or control empty vector for 24 h. The medium was then replaced with fresh complete DMEM media supplemented with M-CSF and RANKL for the duration as specified in the figure legends.

### Animals

The 14-3-3ζ^KO^ (*Ywhaz*^KO^) animals were generated as described before (7). Both WT and *Ywhaz*^KO^ male Lewis rats were maintained in the University of Kentucky vivarium and fed a standard diet. Age-matched (8–12 weeks) littermate Wt and *Ywhaz*^KO^ male Lewis rats were utilized for studies. All animal experiments were conducted in accordance with approved protocol from the Institutional Animal Care and Use Committees of the University of Kentucky.

### BMDM isolation

The cleaned bones of WT and *Ywhaz*^KO^ male rats were used to collect primary bone marrow-derived macrophages (BMDM), as described previously (19). Briefly, the bone marrow was collected by spinning in a clean tube at 1000 rpm for 5 min. The red blood cells were lysed using ammonium-chloride-potassium lysis buffer, followed by plating in the minimum essential medium containing 20% LCM (L929 conditioned media), 15% fetal bovine serum, and 5 mM β-mercaptoethanol. The BMDM were plated at 1 × 10^ˆ6^ per 6-well plate in duplicate, and the M-CSF (10 ng/ml) and RANKL (50 ng/ml) treatments started on day 1. Cells were harvested at 0, 8, 20, and 40 h post-cytokine stimulation and stored in RNA Protect Reagent.

### RNA isolation and quantitative analysis

Total RNA was isolated using TRIzol (Invitrogen), cDNA was prepared using ImProm-II Reverse Transcription Kit (Promega), and the cDNA was analyzed using Radiant SYBR Green PCR mix (Alkali Scientific) in Roche LightCycler 96 instrument and analyzed with the LightCycler 480 Software, v1.5. The expression levels of the mRNAs were normalized to 18S rRNA. For the qRT-PCR analyses of the respective genes, the following primers were used:

rCcl7: ATTCCTCACCCTTGTCCTTTC/CCATTCCTTAGGAGCGACTATTT.

rCcl12: GATCCACATTCGGAGGCTAAA/GTCAGCAGAGAGCTCCTTATC.

rLGR4: CCTTGTGCTTGCCATTTCCT/CCGTGATCAATGGTGCGAAT.

rRAC2: GACTCAGCCATCCATCCTCA/ACAGAGGAGCAGACTTCCAC.

mAcp5: GACGATGGGCGCTGACTTCA/GCGCTTGGAGATCTTAGAGT.

mLGR4: CCTTGTGCTTGCCATTTCCT/CCGTGATCAATGGTGCGAAT.

mCCL7: CATGGAAGTCTGCGCTGAAG/AGAAAGAACAGCGGTGAGGA.

mYwhaz: ACCGTTACTTGGCCGAGGTT/GCAGGCTTTCTCTGGGGAGT.

### Bulk RNA-seq and analysis

The RNA quality and integrity were evaluated by RNA Integrity Number (RIN) analysis using the Agilent RNA 6000 nano kit. The samples with RIN scores of 9 to 10 were used for bulk RNA-sequencing analysis (Novogene Inc). After quality control tests, an mRNA library was constructed using polyadenylate tail capture *via* oligo (dT) beads and then retested for quality control. The library generated was sequenced using the Illumina platform (NovaSeq 6000) with a pair-end 150 base pair sequencing strategy (PE150). Sequencing data obtained from Illumina were transformed into raw sequence reads (raw data) using CASAVA base recognition (base calling). The raw data were filtered for a cleaner downstream analysis. This was done by using the following exclusion criteria: adapter contamination, more than 10% of uncertain nucleotides, and more than 50% of low-quality nucleotides (base quality of <5). The library prepared from the filtered raw reads were mapped to the reference genome_rattus_norvegicus_rnor_6_0_gca_000001895_4. Feature Counts were used to quantify the gene expression of the samples in terms of raw counts. Differential gene analysis was performed using the DESeq2 (v1.20.0) with threshold set as: |log2(FoldChange)| >=1 and Padj ≤ 0.05. The statistical analysis of the DEGs was performed using the following threshold parameters in DESeq2: *p* value ≤ 0.05 and |log2(FoldChange)|>=1. Fragments per kilobase of transcript per million mapped reads (FPKM) cluster analysis was performed to generate heatmaps, clustered using the log2(FPKM + 1) value, with represented values ranging from +1.5 (red, high expression) to −1.5 (blue, low expression). For KEGG enrichment analysis, the clusterProfiler package (v3.8.1) was used, with parameters set to Padj < 0.05. Overall, 14,900 gene transcripts showed significant differences in expression levels. The data, Venn diagrams, heatmaps, and KEGG pathway analysis were produced using Novomagic software from Novogene Inc (https://portal-global.novogene.com/login).

### Immunostaining

The detailed protocol for cellular immunostaining is described elsewhere (52). Briefly, the M-CSF/RANKL-treated primary BMDM were fixed in 4% (v/v) paraformaldehyde, permeabilized with 0.1% Triton X-100, and blocked using 1% BSA. The blocked coverslips were incubated with primary antibodies (LAMP1 and Cathepsin Z) overnight at 4 °C, and the Alexa Fluor-conjugated secondary antibody (Invitrogen Inc) was incubated for 1 h at room temperature, with 3 cycles of washing in between. The coverslips were mounted onto microscopy slides using DAPI-containing VectaShield (Vector Laboratories #H-1200) and imaged using the ECHO Microscope.

### Cell lysis and immunoblots

Cell lysis and Immunoblot analyses were performed using previously described procedures (52). Briefly, the cells were lysed in RIPA buffer containing protease and phosphatase inhibitors (Roche). Total protein extracts were analyzed by Bradford assay for protein content and immunoblot for specific protein detection. Band intensities were measured using NIH Image-J software (https://imagej.net/ij/).

### TRAP staining

TRAP-positive cells were determined using a leukocyte acid phosphate assay kit (Sigma) as per the manufacturer’s instructions. The cells were fixed with 65% acetone, 25% citrate solution, and 8% formaldehyde. The fixed cells were then incubated with the TRAP staining solution in the dark for 1 h at 37 °C. After washing twice with warm water, the cells were counterstained with hematoxylin for 30 s and rinsed with water. TRAP-positive cells that contained three or more nuclei (n > 3) were considered mature osteoclasts when visualized under ECHO microscope.

### Lysosome detection

The lysosomal content was measured in primary BMDM and RAW cells using the dye LysoBrite Red (AAT Bioquest, Inc) according to the manufacturer’s instructions. The dye working solution was prepared by diluting 20 μl of the 500× LysoBrite red with 10 ml of Hanks and 20 mM Hepes buffer. The dye-working solution was added to an equal volume of the cells and incubated (37 °C, 5% CO2 for 30 min). Then, the cells were washed twice with prewarmed (37 °C) Hank's balanced salt solution (HBSS) supplemented with 20 mM Hepes. The MFI was determined at the excitation and emission wavelengths of 532/561 and 585/40 nm, respectively, using the BD FACSymphony A3.

### Statistical analysis

The bulk RNA-seq analysis of WT and *Ywhaz*^KO^ rat BMDMs was performed with two biological replicates and two technical replicates. Each validation experiment with BMDMs is performed with at least four biological replicates and all experiments with RAW cells are performed atleast three times. Exact repeat of each experiment is provided in the figure legends. Quantitative data are shown as mean ± SD. GraphPad Prism (Prism 10) was used to calculate the significance by unpaired Student’s *t* test or two-way ANOVA, depending on the number of sets being examined. Individual details of the test used for analysis are mentioned in the figure legend. Asterisks ∗∗, ∗∗∗, and ∗∗∗∗ show *p*-values of <0.01, 0.005, and 0.001, respectively.

## Data availability

All data presented in this paper are contained within the article. The bulk RNA-seq datasets have been deposited to GEO under the accession record GSE328998.

## Supporting information

This article contains supporting information.

## Conflict of interest

The authors declare that they have no conflicts of interest with the contents of this article.

## Supporting information

Sup.Fig1

Sup.Fig2

Sup.Fig3

Sup.Fig4

Sup.Fig5

Sup.Fig6

Sup.Fig7

Sup.Fig8

Sup.Fig9

Sup.Fig10
