# Supplementary material for: Temporal transcriptomic profiling uncovers multilevel suppression of RANKL-driven osteoclast differentiation by 14-3-3ζ
Source: J Biol Chem. 2026 Aug 24;302(10):113473. doi: 10.1016/j.jbc.2026.113473 (PMC13631583; doi:10.1016/j.jbc.2026.113473)
Supplement: Supplementary file 1 [file mmc1.pdf]

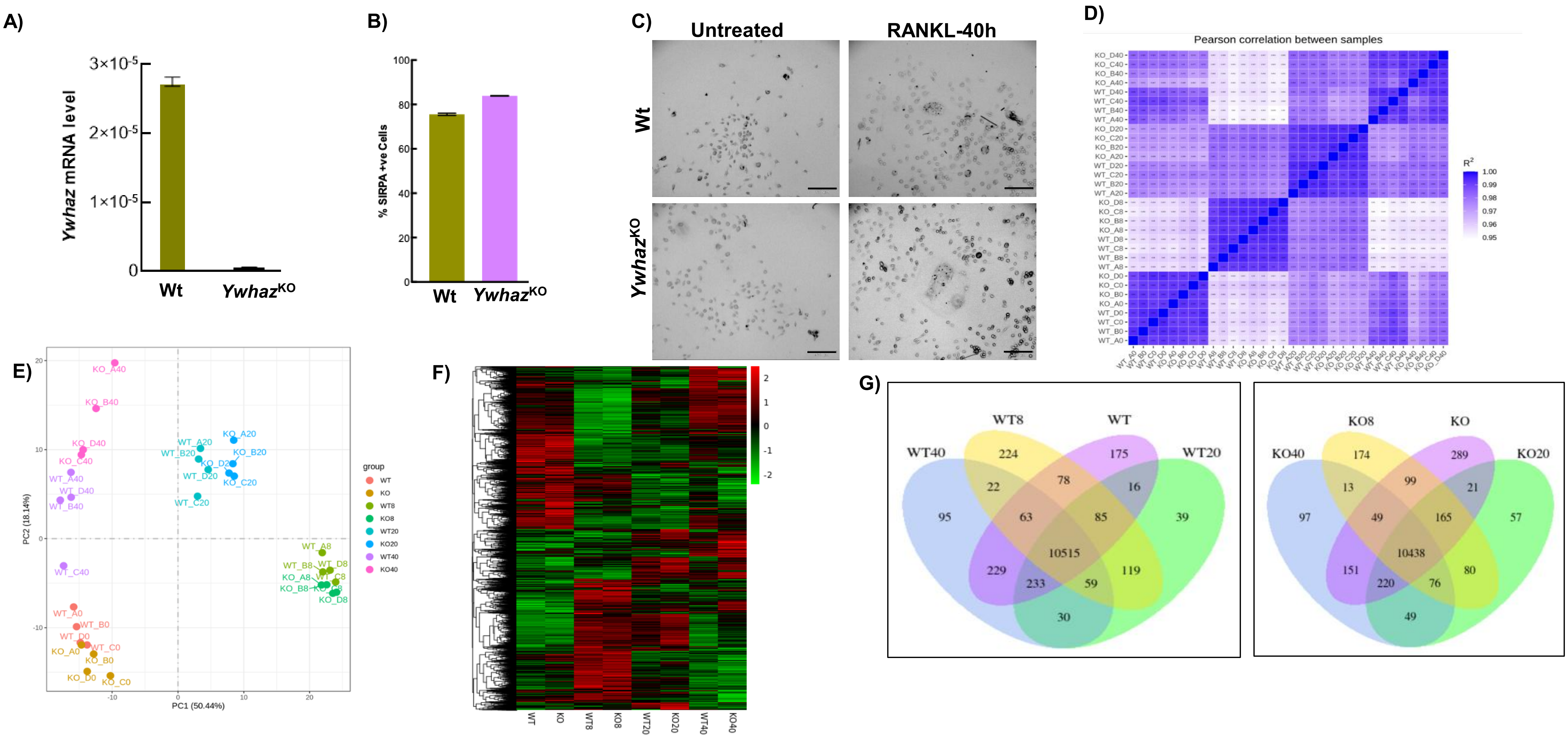

Supplementary Fig 1. A) *Ywhaz* mRNA levels in CRISPR-Cas9 control (Wt) and *Ywhaz*^{KO} RAW cells were analyzed by qRT-PCR (n=2). The expression levels of the mRNAs were normalized to 18S rRNA. B) Primary BMDMs from Wt and *Ywhaz*^{KO} animals were plated overnight and next day, non-adherent cells were stained for surface marker Signal-regulatory protein alpha (SIRPA) to examine the macrophage population by flow cytometric analysis (FACS) (two biological replicates, n=2). C) Primary BMDMs from Wt and *Ywhaz*^{KO} animals were treated with M-CSF/RANKL and examined for cellular adhesions. The scale bars are shown (650 µm). D) Heatmap shows the correlation analysis of differential gene expression levels between the groups. E) The principal component analysis showing the intergroup differences. F) Overall heatmap showing differentially expressed genes from WT and *Ywhaz*^{KO} rat BMDMs treated with or without M-CSF/RANKL indicated time point. G) Venn diagram showing the number of differentially expressed and overlapping genes of WT and *Ywhaz*^{KO} rat BMDMs treated with or without M-CSF/RANKL indicated time point.
