# Supplementary material for: Temporal transcriptomic profiling uncovers multilevel suppression of RANKL-driven osteoclast differentiation by 14-3-3ζ
Source: J Biol Chem. 2026 Aug 24;302(10):113473. doi: 10.1016/j.jbc.2026.113473 (PMC13631583; doi:10.1016/j.jbc.2026.113473)
Supplement: Supplementary file 2 [file mmc10.pdf]

A)

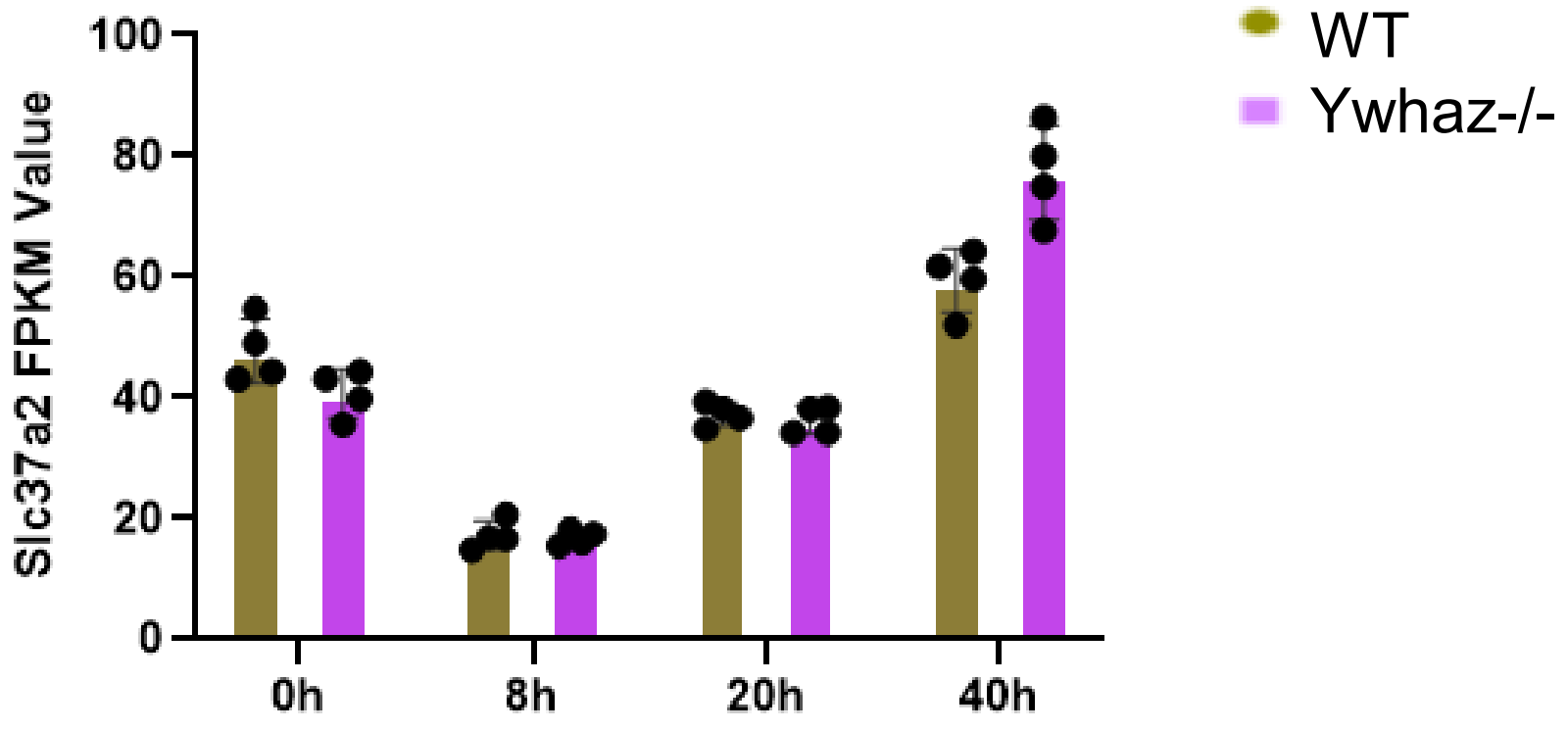

Supplementary Fig 10: The FPKM values of Slc37a2 at the indicated time points in the Wt and *Ywhaz*^{KO} are shown.
