# Supplementary material for: Temporal transcriptomic profiling uncovers multilevel suppression of RANKL-driven osteoclast differentiation by 14-3-3ζ
Source: J Biol Chem. 2026 Aug 24;302(10):113473. doi: 10.1016/j.jbc.2026.113473 (PMC13631583; doi:10.1016/j.jbc.2026.113473)
Supplement: Supplementary file 3 [file mmc2.pdf]

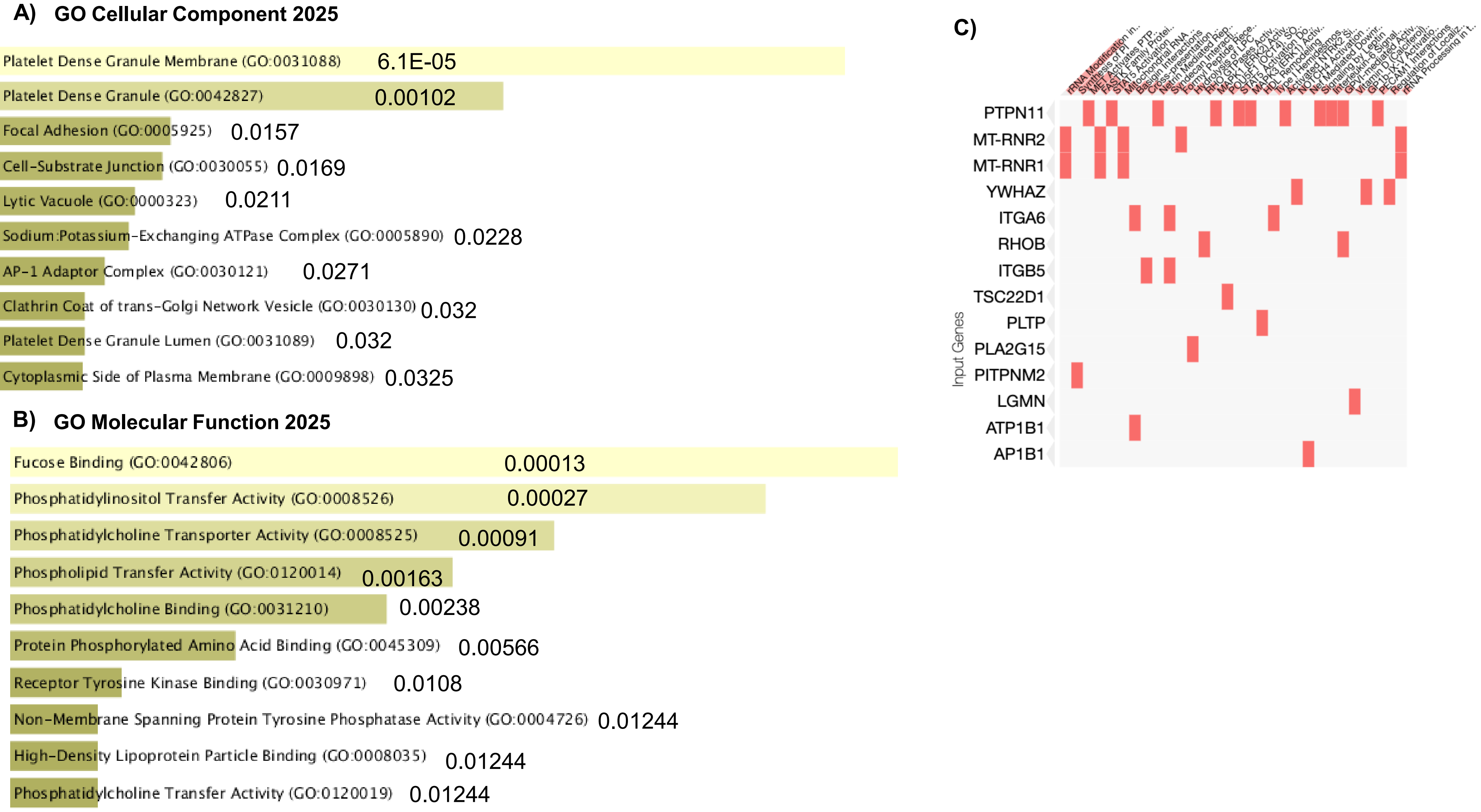

Supplementary Fig 2: A-B). Gene ontology analysis represents the genes are associated with cellular location and different functional categories. C). The reactome pathway analysis shows the genes are enriched in specific pathways. KEGG analyses of selected differentially regulated pathways up and downregulated in *Ywhaz*^{KO} rat BMDMs.
