# Supplementary material for: Temporal transcriptomic profiling uncovers multilevel suppression of RANKL-driven osteoclast differentiation by 14-3-3ζ
Source: J Biol Chem. 2026 Aug 24;302(10):113473. doi: 10.1016/j.jbc.2026.113473 (PMC13631583; doi:10.1016/j.jbc.2026.113473)
Supplement: Supplementary file 4 [file mmc3.pdf]

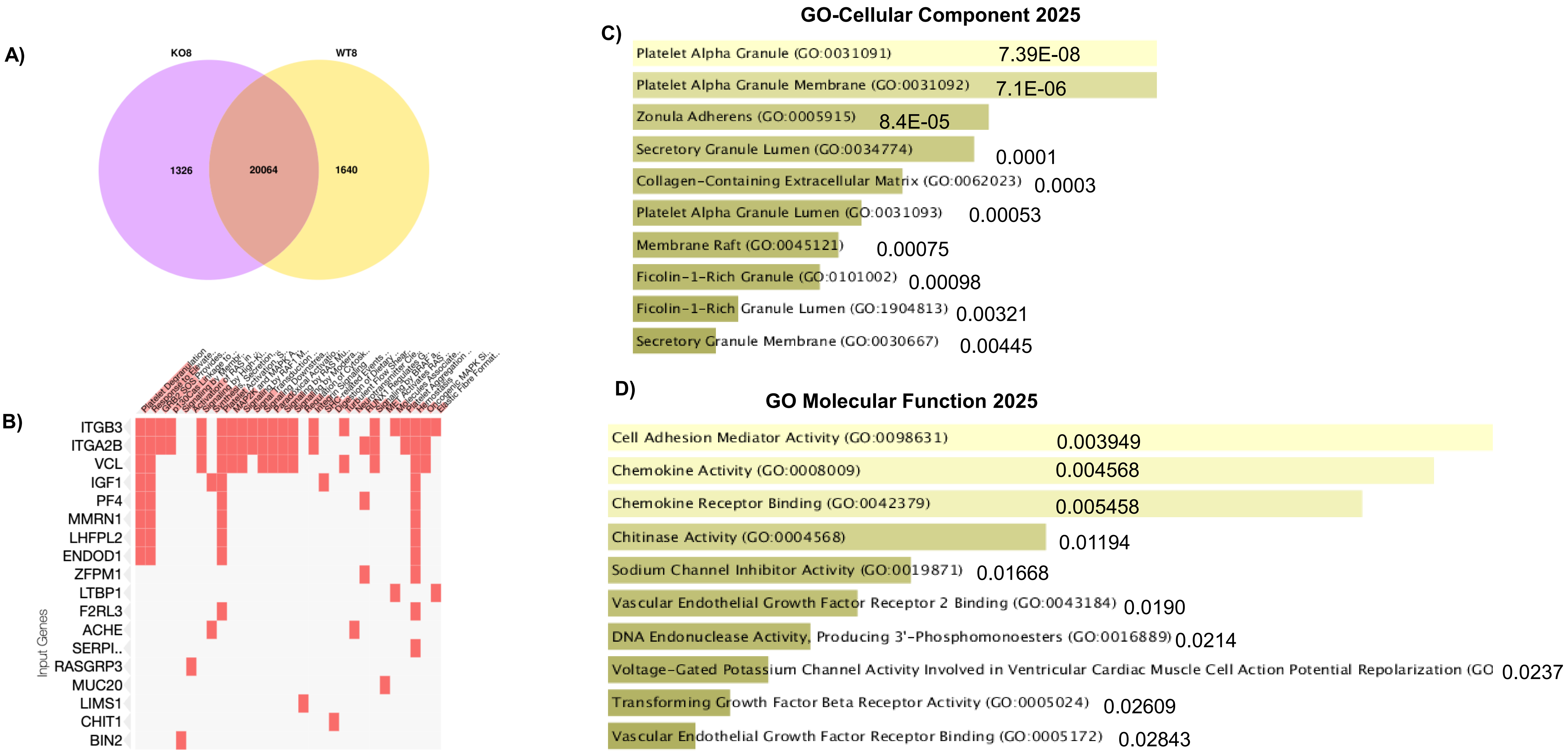

Supplementary Fig 3: A) Venn diagram showing the number of differentially expressed and overlapping genes of untreated and treated WT and *Ywhaz*^{KO} rat BMDMs for 8 h. B) The reactome pathway analysis shows the genes are enriched in the specific pathways. C-D) Gene ontology analysis represents the genes are associated with cellular location and different functional categories.
