# Supplementary material for: Temporal transcriptomic profiling uncovers multilevel suppression of RANKL-driven osteoclast differentiation by 14-3-3ζ
Source: J Biol Chem. 2026 Aug 24;302(10):113473. doi: 10.1016/j.jbc.2026.113473 (PMC13631583; doi:10.1016/j.jbc.2026.113473)
Supplement: Supplementary file 6 [file mmc5.pdf]

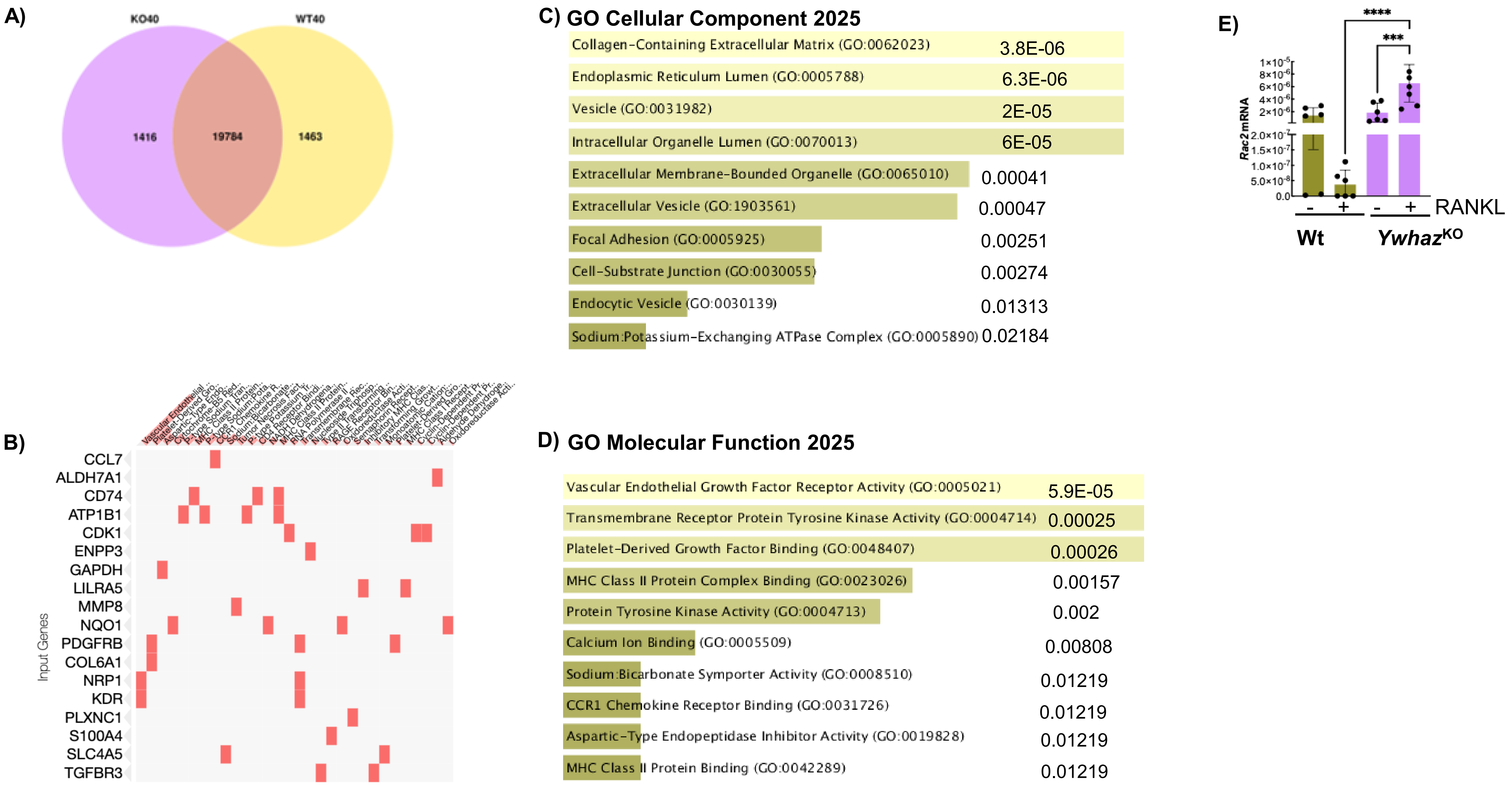

Supplementary Fig 5: A) Venn diagram showing the number of differentially expressed and overlapping genes of untreated and treated WT and *Ywhaz*^{KO} rat BMDMs for 40 h. B) The reactome pathway analysis shows the genes are enriched in specific pathways. C-D). Gene ontology analysis represents the genes are associated with cellular location and different functional categories. E) *Rac2* mRNA levels were analyzed by qRT-PCR in WT and *Ywhaz*^{KO} rat BMDMs treated with or without M-CSF/RANKL for 40 h (n=3). An ordinary one-way ANOVA test was performed for fig. E. The asterisks ***, and **** show p-value of < 0.005, and 0.001, respectively.
