# Supplementary material for: Temporal transcriptomic profiling uncovers multilevel suppression of RANKL-driven osteoclast differentiation by 14-3-3ζ
Source: J Biol Chem. 2026 Aug 24;302(10):113473. doi: 10.1016/j.jbc.2026.113473 (PMC13631583; doi:10.1016/j.jbc.2026.113473)
Supplement: Supplementary file 7 [file mmc6.pdf]

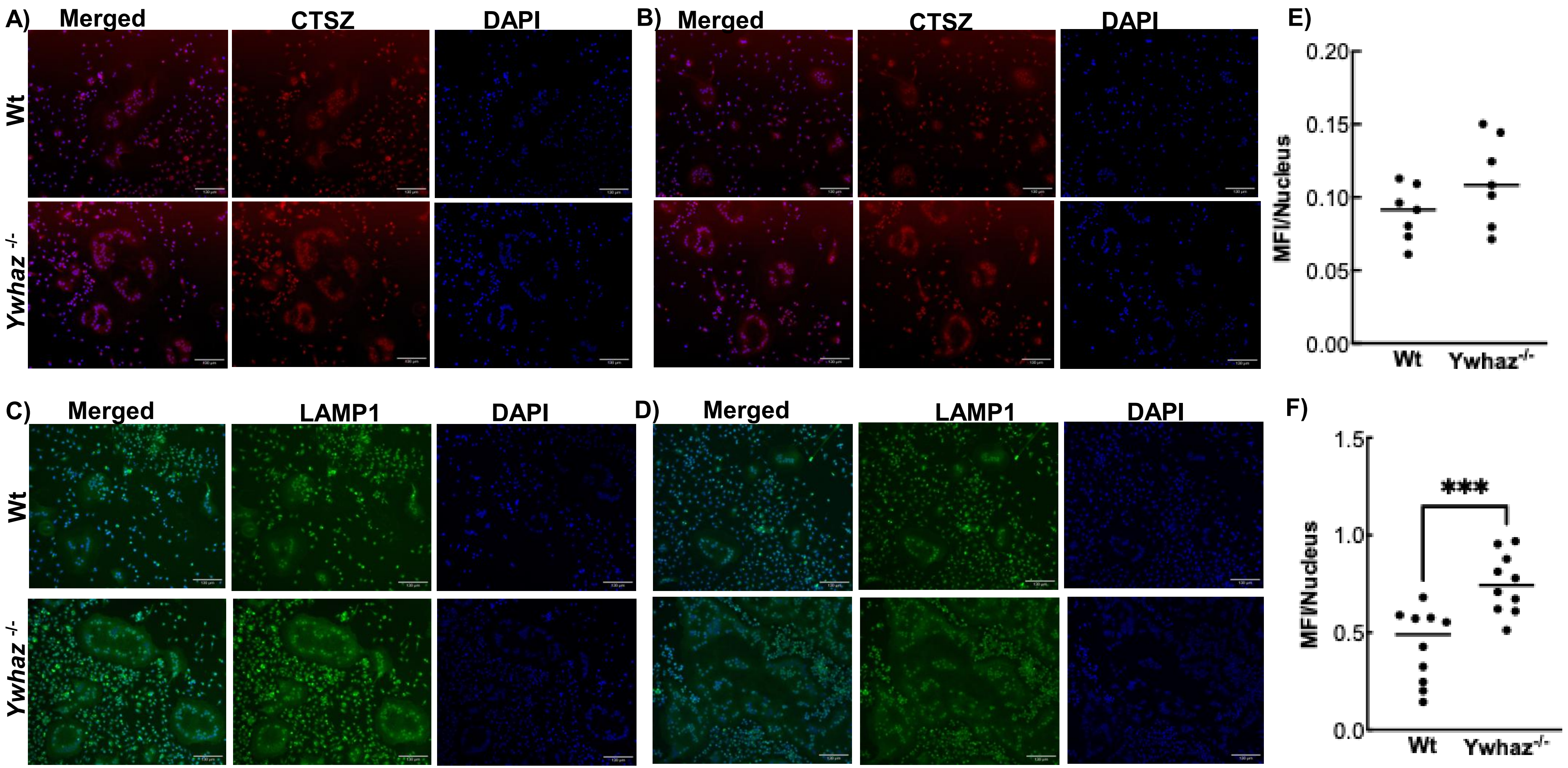

Supplementary Fig 6: A-D) Rat Wt and *Ywhaz*KO BMDMs stained with anti-Cathepsin Z/anti-Lamp1 (n = 5). E-F) The fluorescence intensity normalized to DAPI and plotted using ImageJ from 3-5 microscopic fields each. The scale bars are shown (130μm).
