# Supplementary material for: Temporal transcriptomic profiling uncovers multilevel suppression of RANKL-driven osteoclast differentiation by 14-3-3ζ
Source: J Biol Chem. 2026 Aug 24;302(10):113473. doi: 10.1016/j.jbc.2026.113473 (PMC13631583; doi:10.1016/j.jbc.2026.113473)
Supplement: Supplementary file 8 [file mmc7.pdf]

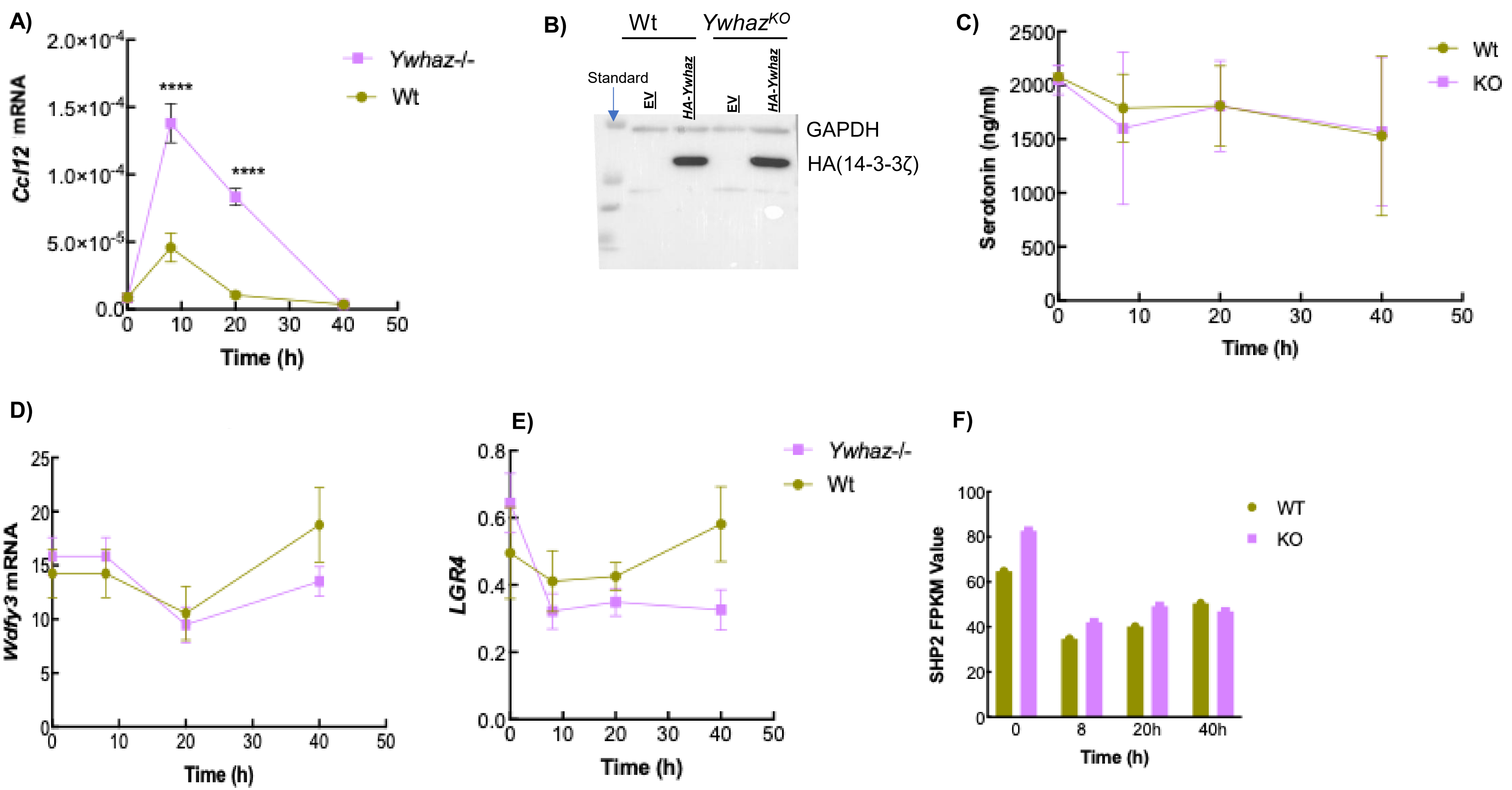

Supplementary Fig 7: A) *Ccl12* mRNA level was analyzed by qRT-PCR in Wt and $Ywhaz^{KO}$ rat BMDMs treated with or without M-CSF/RANKL for indicated time points (n=3). The expression levels of the mRNAs were normalized to 18S rRNA. The two-way ANOVA test was performed for panel A. The asterisks **** show *p*-value of <0.001. B) Expression of HA-14-3-3ζ is shown by immunoblot using anti-HA antibody. Molecular weights are shown on the left of the immunoblot panels. C) Serotonin levels were measured from the condition media of RANKL-treated Wt and $Ywhaz^{KO}$ rat BMDMs for the indicated time points using ELISA (n=2). D-G) The FPKM values obtained for genes *Wdfy3, LGR4* and SHP2 were plotted for the indicated time points as shown in the figure.
