# Supplementary material for: Temporal transcriptomic profiling uncovers multilevel suppression of RANKL-driven osteoclast differentiation by 14-3-3ζ
Source: J Biol Chem. 2026 Aug 24;302(10):113473. doi: 10.1016/j.jbc.2026.113473 (PMC13631583; doi:10.1016/j.jbc.2026.113473)
Supplement: Supplementary file 9 [file mmc8.pdf]

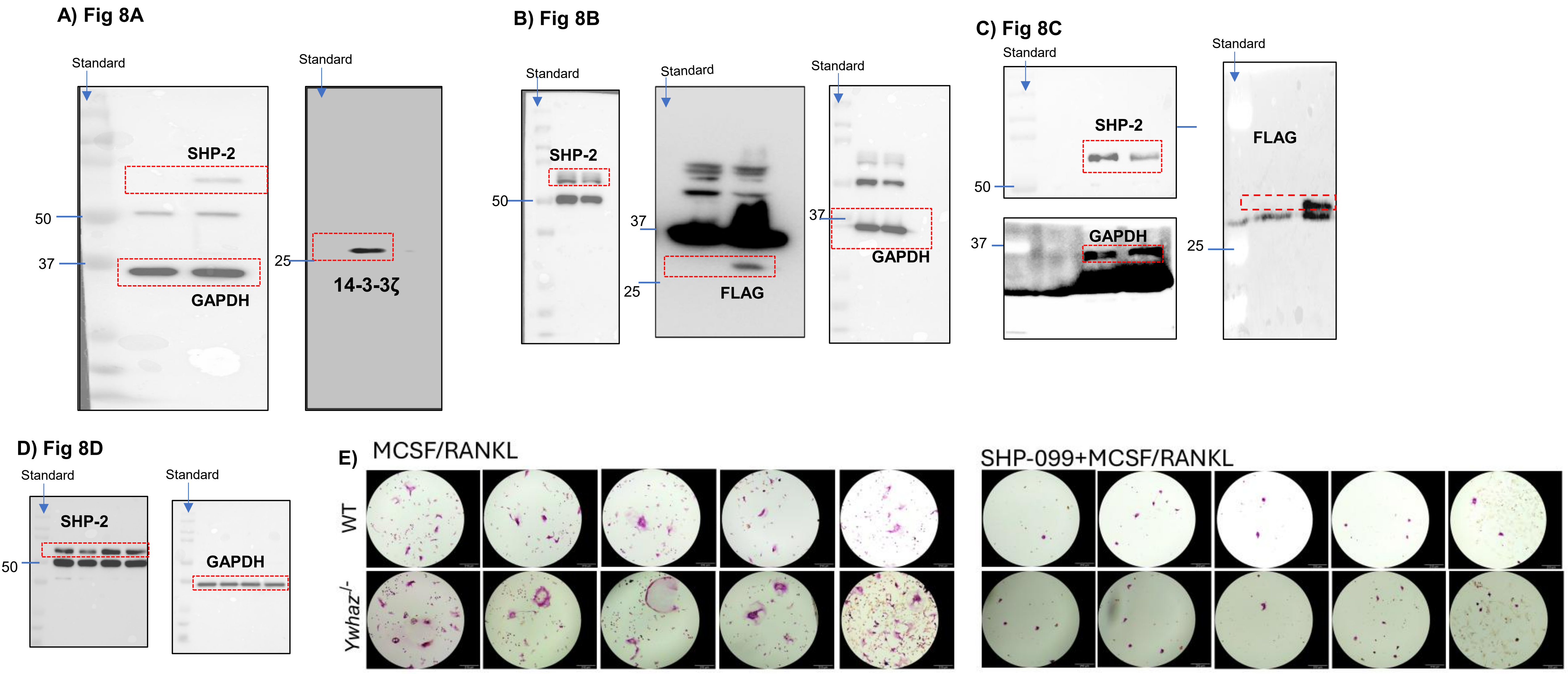

Supplementary Fig 8: A-D) Raw western blots of Fig. 8 are shown. Molecular weights are shown on the left of the immunoblot panels. E) Effect of SHP-2 inhibition on RANKL-induced TRAP+ osteoclasts in Wt and Ywhaz^{KO} BMDM is shown. Representative images with a scale bar of 210 µm are shown.
