# Supplementary material for: Temporal transcriptomic profiling uncovers multilevel suppression of RANKL-driven osteoclast differentiation by 14-3-3ζ
Source: J Biol Chem. 2026 Aug 24;302(10):113473. doi: 10.1016/j.jbc.2026.113473 (PMC13631583; doi:10.1016/j.jbc.2026.113473)
Supplement: Supplementary file 10 [file mmc9.pdf]

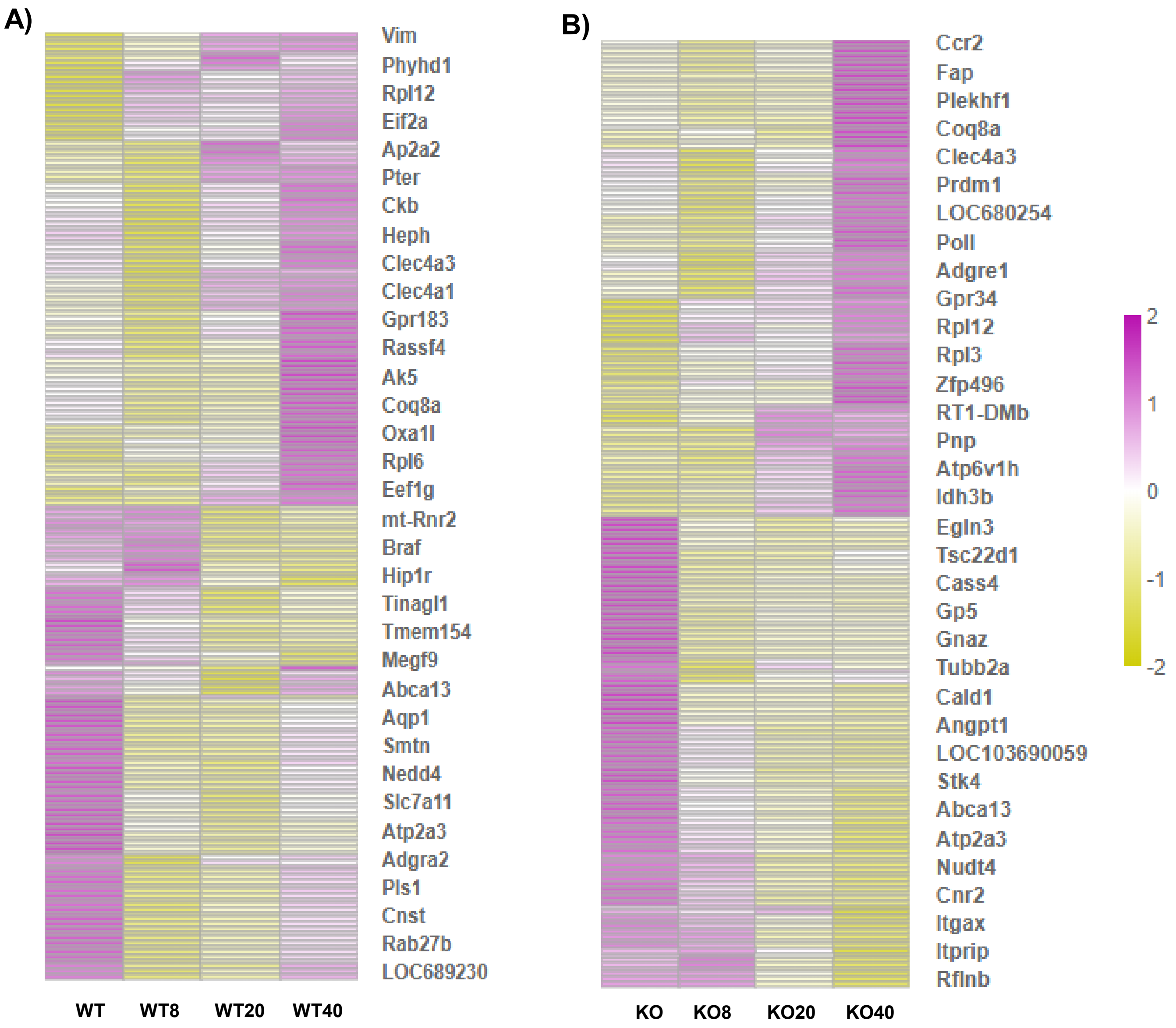

Supplementary Fig 9: A-B). Heatmap showing the top 200 differentially expressed genes from Wt and *Ywhaz*^{KO} rat BMDMs treated with or without M-CSF/RANKL at the indicated time points.
